# A Comprehensive NMR Analysis of Serum and Fecal Metabolites in Familial Dysautonomia Patients Reveals Significant Metabolic Perturbations

**DOI:** 10.3390/metabo13030433

**Published:** 2023-03-16

**Authors:** Stephanann M. Costello, Alexandra M. Cheney, Annie Waldum, Brian Tripet, Maria Cotrina-Vidal, Horacio Kaufmann, Lucy Norcliffe-Kaufmann, Frances Lefcort, Valérie Copié

**Affiliations:** 1 Department of Chemistry and Biochemistry, Montana State University, Bozeman, MT 59717, USA; 2 Department of Neurology, New York University School of Medicine, New York, NY 10017, USA; 3 Department of Microbiology and Cell Biology, Montana State University, Bozeman, MT 59717, USA

**Keywords:** familial dysautonomia, NMR metabolomics, human stool and serum polar metabolite profiles, elongator protein subunit 1, ELP1, metabolism, neurodegenerative diseases, neurological disorders, gut–brain–metabolism axis, multivariate statistical analysis

## Abstract

Central metabolism has a profound impact on the clinical phenotypes and penetrance of neurological diseases such as Alzheimer’s (AD) and Parkinson’s (PD) diseases, Amyotrophic Lateral Sclerosis (ALS) and Autism Spectrum Disorder (ASD). In contrast to the multifactorial origin of these neurological diseases, neurodevelopmental impairment and neurodegeneration in Familial Dysautonomia (FD) results from a single point mutation in the *ELP1* gene. FD patients represent a well-defined population who can help us better understand the cellular networks underlying neurodegeneration, and how disease traits are affected by metabolic dysfunction, which in turn may contribute to dysregulation of the gut–brain axis of FD. Here, ^1^H NMR spectroscopy was employed to characterize the serum and fecal metabolomes of FD patients, and to assess similarities and differences in the polar metabolite profiles between FD patients and healthy relative controls. Findings from this work revealed noteworthy metabolic alterations reflected in energy (ATP) production, mitochondrial function, amino acid and nucleotide catabolism, neurosignaling molecules, and gut-microbial metabolism. These results provide further evidence for a close interconnection between metabolism, neurodegeneration, and gut microbiome dysbiosis in FD, and create an opportunity to explore whether metabolic interventions targeting the gut–brain–metabolism axis of FD could be used to redress or slow down the progressive neurodegeneration observed in FD patients.

## 1. Introduction

The role of the gut–brain axis in neurodegenerative diseases has become increasingly appreciated [1,2,3]. However, the impact of central metabolism dysfunction on the gut–brain axis of neurodegenerative diseases remains poorly understood. Herein, we have investigated serum and fecal polar metabolite changes exhibited in Familial Dysautonomia (FD) patients, and compared these to the levels of polar metabolites measured in healthy family relatives who served as a reference human control group, and are heterozygous carriers of the *ELP1* gene mutation. This comprehensive interrogation of the serum and fecal metabolomes of FD patients is part of our long-term goal to generate new knowledge about the cross-talk between gut microbes, neuronal health, and metabolism in FD [4]. New insights into the gut–brain–metabolism axis of FD could potentially be employed to redress metabolic deficits, and to guide the development of efficacious metabolism-based interventions and treatment strategies aimed at mitigating neurodegeneration and disease progression.

Familial Dysautonomia, also called Riley-Day syndrome or Hereditary and Sensory Autonomic Neuropathy type III, is a debilitating developmental and progressive neurodegenerative disease that occurs primarily in individuals of Ashkenazi Jewish decent, with >99% of all patients sharing the same homozygous founder mutation (c.2204 + 6T > C). FD results from an autosomal recessive point mutation in the *ELP1* gene encoding the elongator complex subunit 1 (ELP1) protein [5,6,7]. This mutation causes inefficient splicing of exon 20, which in turn introduces a premature stop codon, leading to nonsense-mediated decay of the messenger RNA (mRNA) *ELP1* transcript and subsequent tissue specific reduction in ELP1 protein expression [6,7,8]. Although *ELP1* is normally ubiquitously transcribed in all tissues, the protein deficit occurs predominantly in neurons [6,8]. ELP1 functions as a scaffolding protein, for the six-subunit elongator complex that mediates wobble uridine modification of transfer RNAs (tRNAs), and codon-biased regulation of mRNA translation [6,9]. The reduction in ELP1 protein levels decreases translation of mRNAs encoding DNA repair proteins and others involved in histone modification [9]. Reduced ELP1 expression also disrupts mitochondrial integrity and function [10,11], which collectively cause cellular stress and promotes neurodegeneration.

Significant impairments to both the central nervous system (CNS) and peripheral nervous system (PNS) are evident in FD, with the sensory and autonomic nervous systems being most heavily impacted [5,6,12]. Hallmarks and clinical features of the disease include reduced pain and temperature sensation, gastrointestinal dyscoordination and enteropathy, cardiovascular instability, progressive optic neuropathy, proprioception loss, and sudden unexpected death during sleep (SUDS) [5,7]. Unfortunately, there is no cure for FD and the progressive decline in neuronal survival results in a 50% mortality rate of FD patients by the age of 40 [5,6]. Additionally, the quality of life of the patients is devastatingly reduced, with current treatment strategies focusing on symptom mitigation, rather than slowing or redressing disease progression and neurodegeneration [7]. 

FD shares characteristic clinical features with other complex neurodegenerative diseases including Alzheimer’s (AD) and Parkinson’s (PD) diseases, Amyotrophic Lateral Sclerosis (ALS), and neurodevelopmental disorders such as Autism Spectrum Disorder (ASD) [13,14,15,16]. However, because the cause of FD is a single shared genetic mutation [5,6,7], the FD patient population is a more homogenous group to examine than patient cohorts suffering from multifactorial neurodegenerative diseases. Interestingly, mutations, and/or variants, in the genes encoding four of the other elongator complex proteins are associated with neurological and cognitive impairment disorders including ALS (with the *C9orf72* ALS associated gene variant giving rise to reduced ELP3 expression) [17], intellectual disability (associated with a mutation in the *ELP2* gene), and Rolandic epilepsy or ASD (associated with a mutation or microdeletion, respectively, in the *ELP4* gene) [13,18]. 

Characterizing the metabolite profiles of serum and stool samples collected from FD patients offers an opportunity to generate insights into metabolic pathways that are impacted at the systemic level and/or that are influenced by gut microbial activity. Given the rich opportunity that the serum and stool metabolomics data present for gaining new knowledge about molecular networks underlying the FD disease state, we sought to expand upon our analysis of the gut–brain–metabolism axis in FD recently reported by our group [4], by evaluating our proton (^1^H) nuclear magnetic resonance (NMR)-based serum and fecal metabolome data in a stepwise population and pairwise manner. This comprehensive analysis ensures that noteworthy metabolite patterns relevant to FD are not overlooked, and provides an extended list of metabolites of interest, in addition to the subset originally identified as being significant when using a strictly statistical, random permutation test [4]. Examination of the full range of serum and fecal metabolite changes has provided insightful information about metabolic pathways associated with the gut–brain–metabolism axis in FD that were not readily apparent in our initial study.

Herein, an untargeted one dimensional (1D) ^1^H NMR metabolomics approach was employed to characterize the polar metabolite profiles of serum and stool samples obtained from FD patients and their matched relatives (who served as a control group) who are heterozygous carriers for the FD mutation. The present study has uncovered additional, prominent changes in the serum and fecal metabolomes of FD patients compared to those of their healthy relatives, expanding upon those reported in [4]. The significance of these findings, with respect to the gut–brain–metabolism axis of FD and its potential relevance to other neurological diseases, is discussed.

## 2. Materials and Methods

### 2.1. Participants and Sampling Scheme

FD patients and their relatives were recruited through the Dysautonomia Center at New York University (NYU) Langone Health. Recruitment for this study began in 2016 and samples used for this metabolite profiling analysis were collected during the patients’ clinical visits from September of 2016 through January of 2020. A total of 104 participants were enrolled and involved 50 FD patients and 54 relatives (Supplementary Table S1). Worldwide, there exist only 350 individuals diagnosed with FD to date; the 50 patients recruited for this study thus represent 14.30% of the total population of individuals with this disease, which is a significant percentage. All patients enrolled in the study were homozygous for the FD founder mutation in the *ELP1* gene (c.2204 + 6 T > C) and all relatives were heterozygous, including one sibling who was enrolled as a healthy relative control. Individuals who are heterozygous for the *ELP1* gene mutation do not exhibit any of the FD clinical phenotypes and are neurotypical. Of all enrolled patients, 42 had a corresponding paired relative also enrolled; 8 FD patients were enrolled, but had no samples provided by a relative. Of the 42 FD patients enrolled with a relative, 11 of those were enrolled with an additional relative (i.e., mother or father) and 1 FD patient was enrolled with a mother, father, and sibling. The total number of participants included 45 males and 59 females. Of these, 21 male and 29 female FD patients were represented (Supplementary Table S1). 

In 2016 and 2017, only stool samples were collected from the original cohort of 22 FD patients and matched control relatives. In 2018, serum samples and biological replicates were collected, together with additional stool samples from newly enrolled participants. Ideally, stool and serum samples were obtained from every participant, though individual comfort level and ability to donate prevented this in some instances. Additionally, biological replicates were obtained at times, although not very often; only 1 biological replicate at a time was included in any given statistical analysis. A summary of patient and control relative samples provided are listed in Supplementary Table S1. Clinical data (Blood Urea Nitrogen (BUN), Creatine, BUN: Creatine, estimated Glomerular Filtration Rate (eGFR), Alanine Aminotransferase (ALT), Aspartate Transaminase (AST), AST:ALT and Alkaline phosphatase levels) were provided for patients’ samples that were de-identified at times of sample collections, and are included as Supplementary Information (Supplementary Figures S1 and S2).

### 2.2. Blood Serum and Stool Sample Collection

The Dysautonomia Center at NYU Langone Health collected and provided all samples used in this study. Due to the fragile nature of some of the patients’ health, and to mitigate additional stress that fasting could have induced on the patients, no sample donors were required to fast prior to any sample collection. Peripheral venous blood samples were collected in test tubes containing clot activator, allowed layer separation for 30 min, and spun at 3000 rpm for 10 min for serum separation. Aliquots of serum were partitioned into 15 mL vials and frozen at −20 °C, batch shipped overnight on dry ice and stored at −80 °C until used for metabolomics analysis. Collection kits (commode/hat) were provided to participants for self-collection of stool samples at home or in-patient visits. Stool samples were collected at the NYU Langone’s Dysautonomia Center, transferred to 50 mL conical tubes either fresh, from the same day, or previously frozen at −20 °C by the patient and/or relative. As with the serum samples, stool samples were stored at −20 °C at the clinic until batch shipped overnight on dry ice and then stored at −80 °C until they were extracted for metabolomics analysis. 

### 2.3. Human Serum Sample Preparation and Serum Metabolite Extraction

Serum samples stored at −80 °C were thawed on ice, partitioned into 400 µL aliquots and 1600 µL of acetone added to each aliquot (1:4 *v*/*v* ratio of serum to acetone). Samples were mixed by inversion, incubated at room temperature (25 °C) for 20 min, and then incubated at −20 °C for 1 h. Samples were centrifuged at 10,000× *g* for 10 min and resulting supernatants were transferred to new 2.0 mL microcentrifuge tubes, and dried using a speed vacuum concentrator overnight, without heat. The dried metabolite mixtures were reconstituted in 600 µL of serum-specific NMR buffer (0.25 mM sodium trimethylsilylpropanesulfonate (DSS) in 90% H_2_O/10% D_2_O, 0.4 mM imidazole, 25 mM NaH_2_PO_4_/Na_2_HPO_4_, pH 7), and transferred to a 5 mm Bruker NMR tube. 

### 2.4. Human Fecal Sample Preparation and Stool Metabolite Extraction

Fecal samples stored at −80 °C were thawed at room temperature (25 °C) and homogenized by spatula stir mixing in their original 50 mL conical tubes. Aliquots of 500 mg were measured into 2 mL twist cap centrifuge tubes, flash frozen and stored at −80 °C until further use. For each aliquot, 1 mL of stool-specific NMR buffer (0.1 M Na_2_HPO_4_/KH_2_PO_4_, pH 7.37, 0.25 mM DSS, 0.4 mM imidazole, 0.01% sodium azide, in 90% H_2_O/10% D_2_O, pH 7.0) was added to achieve a 1:2 *w*/*v* (wet fecal-weight to buffer-volume) ratio. Samples were homogenized using a Millipore FastPrep-24^TM^ 5G bead beater, set at 6.0 m/s for 40 s. Samples were centrifuged at 10,000× *g* for 10 min and the supernatants were transferred to new 2.0 mL microcentrifuge tubes. The stool metabolite extraction process was repeated once more using the centrifuged fecal pellets and a fresh 1 mL of stool NMR buffer to collect a second supernatant fraction for each sample. Both supernatant fractions were combined, centrifuged, and filtered using the following: centrifugation at 21,000× *g* for 10 min and filtering using a 4 µm syringe filter. Resulting filtrates were then centrifuged at 21,000× *g* for 10 min and filtered using a 3 kDa filter to remove residual large particulates. The final pH of the sample was adjusted to 7.0, prior to transferring 600 µL of the sample solution into a 5 mm Bruker NMR tube for NMR experiments. A second 500 mg aliquot of stool sample was lyophilized to determine the percent water weight and dry mass for each extracted metabolite stool aliquot. 

### 2.5. NMR Spectra Acquisition and Preprocessing

All NMR spectra were collected using a Bruker 600 MHz (^1^H Larmor frequency) AVANCE III solution NMR spectrometer equipped with a 5 mm triple resonance (^1^H, ^13^C, ^15^N), liquid helium-cooled cryoProbe, automatic sample loading system (SampleJet), and Topspin software (Billerica, MA, USA, Bruker version 3.2). The Bruker gradient-based water suppression “zgesgp” pulse sequence [19,20] was used for the acquisition of 1D ^1^H NMR spectra, which were recorded at 300 K with the following parameters: 256 scans, 64K data points, a 69 µs dwell time, and a ^1^H spectral window of 12.0166 ppm, resulting in a NMR spectrum data acquisition time period of ~4.5 s. A recovery delay time (D1) between acquisitions was set to 2.0 s, resulting in a total relaxation recovery delay of ~6.5 s between scans. Chemical shift referencing using DSS and phase correction of 1D ^1^H NMR spectra were conducted using Topspin (Billerica, MA, USA, Bruker version 3.2), as previously reported [19,21,22]. Baseline and phase correction were executed in Topspin with 0.80 Hz line broadening for exponential multiplication prior to Fourier transformation to give the preprocessed ‘1r’ NMR file. 

### 2.6. NMR Spectra Profiling and NMR Signal and Metabolite Annotation Validation

Chenomx NMR Suite software (version 8.4; Chenomx Inc., Edmonton, AB, Canada) was used for the additional processing of 1D ^1^H NMR spectra and metabolite identification (i.e., metabolite ID) and quantification. The preprocessed ‘1r’ NMR spectral files were imported into the Chenomx software and baseline adjusted using the automatic cubic spline function (Chenomx Spline). Manual breakpoint adjustments were applied to obtain a well-defined, flat baseline that did not bias the area under the spectral signal intensities, following guidelines from Chenomx application notes and previously reported methods [23,24]. The most upfield ^1^H signal of DSS was set to 0.0 ppm for chemical shift referencing, and the ^1^H NMR resonance of imidazole was used to correct for small chemical shift variations arising from slight changes in sample pH. Metabolite identification and quantitation was achieved by fitting the spectral patterns present in reference spectra of small molecules, which are part of the Chenomx small molecule spectral database for 14.1 T magnetic field strength NMR spectrometers and the human metabolomics database (HMDB) [25], to the ^1^H chemical shifts, spectral splitting patterns, and signal intensities present in each stool or serum spectrum. A manual peak-based fit style in Chenomx was applied, which permitted optimal fits for compound peak cluster location and intensity [26]. Characteristic NMR spectra of serum and stool samples, with annotated metabolite IDs, are presented as illustrations in the Supplementary Figures S3 and S4, respectively. Relative intensities were calibrated to DSS (0.25 mM), which was used as an internal standard to quantify metabolite levels and to generate lists of metabolite concentrations. Confirmation of the metabolites’ IDs was achieved by spiking of the pure metabolite standards into the metabolite mixture samples, as needed. 

### 2.7. Unpaired Univariate and Multivariate Statistical Analyses 

Quantitative metabolite profiles were exported from the Chenomx software, as µM concentrations. Prior to multivariate statistical analyses using MetaboAnalyst v.4.0 (Edmonton, AB, Canada), the metabolite concentrations were normalized to serum volume (nanomoles/mL) and to dry fecal mass (nanomoles/g) for stool samples [27]. The serum metabolite concentrations varied widely, from <10 nmol/mL for some metabolites to >5000 nmol/mL for others (Supplementary Table S2). Stool metabolites also varied in concentrations, ranging from <5 nmol/g to >200,000 nmol/g and varied between and within the sample populations as seen in the standard deviations reported in Supplementary Table S3.

The biomass normalized data were evaluated in terms of percent differences in mean metabolite concentrations between the FD patient group and the healthy relative control group. For multivariate and univariate statistical analyses, metabolite concentrations were statistically normalized using logarithmic transformation (log base 10) to ensure a Gaussian distribution of the data [28] and autoscaling, (i.e., centering of each metabolite concentration around the mean divided by the standard deviation) [29]. The data were then analyzed using unsupervised Principal Component Analysis (PCA), supervised Partial Least Squares-Discriminant Analysis (PLS-DA), random forest, and volcano plot analyses (*t*-tests and fold changes (FC)). Additional validation metrics of the PLS-DA models were applied using MetaboAnalyst v.4.0 package and associated R programs. PLS-DA model validity was assessed using a leave-one-out validation test for predictive variability metrics (Q^2^) and predicted residual sum of squares (PRESS) error (R^2^), separation distance and prediction accuracy permutation tests (*n* = 1000), classification error rates (CER), and area under the receiver operating characteristic curve (AUROC) (Supplementary Figures S5 and S6) [30]. 

### 2.8. Paired Multivariate Statistical Analyses

To account for variability observed in FD patient serum and stool metabolite profiles, paired analyses were conducted by pairing the metabolite profiles of an FD patient to those of their direct healthy relative(s), who served as control(s). Two lists (referred to as versions 1 and 2) of FD patient-healthy relative pairs were generated, using the following exclusion criteria: (1) data from an FD patient or a relative were omitted if there was no sample to be paired with, i.e., when a patient or relative did not donate a sample with their family member; or (2) when an additional family member donated a sample, resulting in two or more pairing schemes; and (3) biological replicates (i.e., the same donor provided a sample more than once). These exclusion criteria ensured that samples were always paired and that an individual was only represented once in the dataset by omitting multiple patient–relative pairings and biological replicates. Altogether, this led to the generation of 34 possible unique patient–relative pairs for a given paired dataset, which were used for the paired analysis of either serum or stool metabolite patterns.

In the end, two randomly generated paired datasets were used (referred to as paired versions 1 and 2) and the metabolite patterns were examined for similarities and differences between the FD patient and their healthy relative control. These two lists also enabled us to evaluate whether the metabolites found to exhibit significant level differences between the FD patient and relative, depended on which version of the pairs was employed. Similar to our unpaired data analyses, paired *t*-tests and FC analyses were conducted using MetaboAnalyst v.4.0 (Edmonton, AB, Canada) and evaluated using volcano plots.

### 2.9. Metabolic Pathway Impact Analysis

Once sets of metabolites were identified from the serum and stool metabolomics data and found to be significant with statistical analyses, metabolite patterns were further investigated for their relevance to both human and gut-microbial metabolic pathways. A literature review was first conducted to mine published reports about established pathway(s) and function(s) associated with each individual metabolite or class of metabolites. A metabolic pathway impact analysis was then performed using MetaboAnalyst v.4.0 (Edmonton, AB, Canada) to ensure that all possible pathways of interest were taken into account or not excluded due to a potential lack of available information in the literature. 

## 3. Results

### 3.1. Metabolite Concentration Differences Separate FD Patients from Their Healthy Control Relative Populations

A total of 55 serum and 73 stool polar metabolites were identified and quantified from the analysis of 1D ^1^H NMR spectra obtained from FD patients and control relatives, using the Chenomx 8.6 (Chenomx Inc., Edmonton, AB, Canada) software suite. 

Percent differences in metabolite mean concentrations (FD patient/Relative control) were calculated to identify potential metabolites with concentration differences that contribute to group separation between the FD patient and healthy relative (Supplementary Tables S2 and S3) populations. We determined that metabolite level differences < 10% were likely not significant due to the extensive heterogeneity of FD disease phenotypes. Serum metabolites with percent level differences >10% were viewed as potentially informative of serum metabolome changes between patients and controls, and were examined as possible contributors to the distinction between the FD patient and healthy control groups, while mean metabolite level differences ≥ 40% were viewed as potentially more discriminatory. A similar classification of fecal metabolites based on percent differences in mean concentrations was conducted. However, a threshold of > 20% difference was used to exclude select stool metabolites for which level differences were most likely too small to discriminate between FD patients and healthy controls, as the heterogeneity of the stool metabolite profiles was higher than the one observed for the serum samples.

Using this approach, 22 serum metabolites were selected as potentially valuable discriminators of the FD disease state (Supplementary Table S2) from the healthy relative control group. Serum metabolites exhibiting ≥ 40% mean differences between the FD patient and control groups included: xanthine, 1,7-dimethylxanthine, urea, and π-methylhistidine. Six serum metabolites with mean percent differences ranging between 20% and 40% included: glycolate, propylene glycol, dimethyl sulfone, methanol, glycine, and 2-hydroxybutyrate. Lastly, 12 serum metabolites exhibited 10% to 20% mean concentration differences, and included the following: isopropanol, inosine, betaine, pyruvate, dimethylamine, glycerol, serine, leucine, valine, taurine, isoleucine, and alanine. 

Similarly, 21 stool metabolites with a mean percent difference ≥40% were identified and included: dopamine, lactate, formate, beta-alanine, choline, malonate, ethanol, succinate, 5-aminopentanoate, 4-hydroxyphenylacetate, 1,1-dimethylbiguanide, tyramine, taurine, glucitol, ornithine, histidine, trimethylamine, sarcosine, isocaproate, p-cresol, and tryptophan (Supplementary Table S3). Stool metabolites with a mean percent concentration difference between the FD patients and healthy controls ranging between >20% and <40% included: glycine, indole, 2-aminobutyrate, valine, glycerophosphocholine, isovalerate, threonine, phenylalanine, orotic acid, alanine, D-glucose, glutarate, methylsuccinate, lysine, leucine, phenylacetic acid, isoleucine, arginine, xylose, proline, tyrosine, and isobutyrate (Supplementary Table S3). 

### 3.2. The Serum and Stool Metabolomes of the FD Patient Group Differ Significantly from the Metabolomes of the Healthy Relative Control Group 

**Figure 1:**
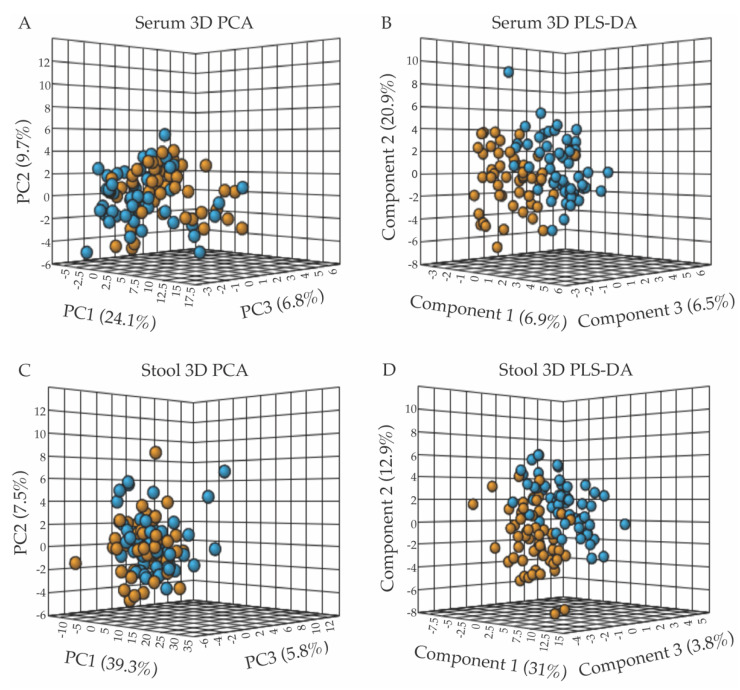
Multivariate statistical analyses of serum and fecal metabolic profiles separate the FD patient population (orange) from the control relative group (blue), with panels (**A**,**B**) presenting the 3D-PCA and 3D-PLS-DA scores plots from the analyses of serum metabolite patterns, and panels (**C**,**D**) those from fecal sample analyses. (**A**) 3D-PCA scores plot obtained from the principal component analysis (PCA) of serum metabolite patterns reveals little separation between the FD patients and healthy control relative groups, with PC1, 2, and 3 accounting for 40.6% of the variance. (**B**) 3D-PLS-DA scores plot obtained from partial least-squares discriminant analysis (PLS-DA) of serum metabolites resulted in the separate clustering of the FD patient and healthy relative control groups, with components 1–3 accounting for 34.3% of the variance. (**C**) 3D-PCA scores plot of stool metabolite profiles, revealing minimal separation between groups, with PC1-3 accounting for 52.6% of the variance. (**D**) 3D-PLS-DA scores plot analysis of stool metabolite profiles indicated a clear separation of the FD patient group from the control group based on distinct fecal polar metabolomes, with components 1–3 accounting for 47.7% of the variance. All data shown in Figure 1 (3D-PCA and PLS-DA scores plots) were generated using the MetaboAnalyst v.4.0 software (Edmonton, AB, Canada).

Metabolite concentrations normalized to volume (for serum samples) or dry weight (for stool samples) were further normalized for statistical analyses using the MetaboAnalyst software v.4.0 (Edmonton, AB, Canada) [27] by applying the log transformation and autoscaling functions. A population-wise assessment of the metabolome profiles of the FD patient and control groups was conducted using an unpaired, unsupervised multivariate principal component analysis (PCA), to assess whether the two human subject groups could be separated based on distinct serum or stool polar metabolomes. A three-dimensional (3D) PCA scores plot of the serum metabolite profiles, shown in Figure 1A, revealed a small but noticeable clustering of the FD patient group (orange) from the healthy relative control (blue) group. The extent of the separation of the two groups, based on characteristic serum metabolite profiles, was further examined using a supervised partial least squares-discriminant analysis (PLS-DA) (Figure 1B) [31]. Although the 3D-PCA scores plot of the stool metabolomes displayed little separation between the FD patient and control groups (Figure 1C), the PLS-DA supervised analysis was more informative, revealing a clearer separation between the two groups (Figure 1D). Examination of the scree plots associated with the 3D-PCA analyses provided information regarding the contribution of each principal component (PC) to the total variance, and demonstrated that PC1-3 are most influential (in terms of their % contribution to the total variance) for the separate clustering of the FD patient group from the healthy relative control group (Supplementary Figure S7).

To assess the legitimacy of the PLS-DA models, validation metric analyses, including R^2^, Q^2^, permutation tests, CER, and AUROC analyses were performed and are included as Supplementary Information (Supplementary Figures S5 and S6) [30]. Results from these validation tests demonstrated the validity of the PLS-DA models, and indicated that the separation of the FD group from the control group based on distinct serum and fecal metabolomes is a real phenomenon, and that the PLS-DA models are not overfit. Some of the validation metrics examined were at what we may consider the validation limit (for example, representing a 25% CER; see Supplementary Information), but within the range of values expected given the nature and variability of the serum and stool samples received, and the fact that the patients and relatives provided serum and stool samples at their convenience rather than at fixed times and were not diet restricted. 

Random forest analyses of serum and stool metabolite profiles further supported the separate clustering of the FD and control groups and were consistent with CER results associated with PLS-DA models (Supplementary Figure S8). 

**Figure 2:**
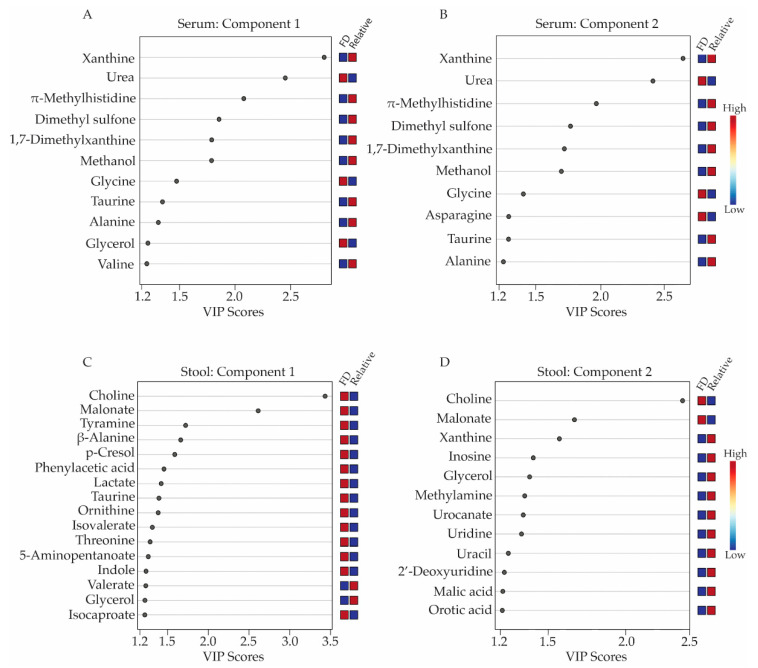
Variable importance in projection (VIP) score plots corresponding to the first two components of the 3D-PLS-DA models obtained from serum and stool metabolite profiles. Metabolites with VIP scores ≥ 1.2 were considered a significant contributor to the distinct clustering of the FD patient and the healthy relative control groups along components 1 and 2 of the 3D-PLS-DA scores plots. Panels (**A**,**B**) display the VIP score plots for the serum metabolites, while panels (**C**,**D**) presents the VIP score plots for the stool metabolite profiles. Relative metabolite levels range from high (red boxes) to low (blue boxes), indicating whether the metabolite concentrations are higher or lower in the FD patient group relative to the levels measured in the healthy relative control group. All VIP scores plots shown in Figure 2 have been generated from PLS-DA modeling conducted in MetaboAnalyst v.4.0 (Edmonton, AB, Canada).

Metabolites with concentration differences that contributed most significantly to the separate clustering of the FD patient and healthy relative control groups in the 3D-PLS-DA analyses were further evaluated using variable importance in projection (VIP) score plots (Figure 2), which provided valuable information on useful metabolite discriminators when associated with VIP scores ≥ 1.2 [22]. VIP Scores for component 1-3 (Figure 2 and Supplementary Tables S4 and S5) were examined in terms of which specific metabolites contributed most to the group separation along component 1, 2, or 3. Not surprisingly, several metabolites contributed to group separation along more than one component axis of the 3D-PLS-DA scores plots shown in Figure 1B,D. 

When examining serum metabolites associated with components 1 and 2 of the PLS-DA scores plots, the VIP scores plots identified 11 and 10 serum metabolites, respectively, that were significant contributors (i.e., VIP ≥ 1.2) to the separate clustering of the FD patient and control groups (Figure 2A,B, and Supplementary Table S4), with glycerol and valine being unique to component 1 and asparagine being unique to component 2. Seven of these metabolites were lower in concentrations in the sera of FD patients and included: xanthine, π-methylhistidine, dimethyl sulfone, 1,7-dimethylxanthine, methanol, taurine, alanine, and valine, while four metabolites, urea, glycine, glycerol, and asparagine were higher in concentration in the FD patient group compared to the healthy relative control group. 

For the stool samples, 17 metabolites with VIP scores ≥ 1.2 were identified. For component 1 (Figure 2C, Supplementary Table S5), 14 of these were in higher concentration in the stool of FD patients and included choline, malonate, tyramine, beta-alanine, p-cresol, phenylacetic acid, lactate, taurine, ornithine, and isocaproate. Two remaining metabolites with a VIP ≥ 1.2 were lower in concentrations in the stool of the FD patients and consisted of valerate and glycerol. Additionally, the VIP scores plot corresponding to component 2 of the PLS-DA model highlighted 10 other stool metabolites that were important for the distinct classification of the two human subject groups and included xanthine, inosine, methylamine, orotic acid, malic acid, urocanate, uracil, 2′-deoxyuridine, uridine and methionine, all exhibiting lower concentrations in the stool samples of FD patients compared to healthy relative controls, except for methionine (Figure 2C and Supplementary Table S5). 

Further evaluation of the metabolome differences between the FD patient and healthy relative control groups was performed using an unpaired volcano plot analysis, which takes into account results from both two-sample *t*-tests and fold change (FC) analyses. The unpaired volcano plot analysis (Table 1) identified six significant serum metabolites (false discovery rate (FDR) corrected *p* value < 0.05 and FC threshold ≥ 1.2) consisting of xanthine, urea, π-methylhistidine, 1,7-dimethylxanthine, dimethyl sulfone and methanol, which were all lower in concentrations in the FD group except for urea (Table 1). When using an FDR corrected *p* value ≤ 0.05 and a fold change threshold of ≥2.0, only two stool metabolites, choline, and malonate, were found to be significant in the unpaired data analysis. When statistical stringency was relaxed (i.e., using an FDR corrected *p* value ≤ 0.3 and FC ≥ 1.2), three additional stool metabolites, tyramine, beta-alanine, and p-cresol were identified as significant, and all five metabolites were higher in concentrations in the stool samples of FD patients compared to the control group (Table 1).

### 3.3. Paired Analyses of FD Patient and Healthy Relative Serum and Stool Metabolomes Further Highlights Metabolic Changes Associated with FD

Next, we conducted paired analyses of the serum and stool metabolite profiles to account for individual patient to relative control pair variability. Pairwise analyses of FD patient-healthy relative pairs were undertaken to examine whether additional metabolite patterns that report on FD could be revealed, and which could have been masked in the population-wise studies due to distinct external factors affecting FD patients or their relatives. These potentially confounding factors could be due to FD patients or relatives experiencing different environments, being on different diets, or in the case of FD patients, exhibiting different disease severity traits. 

Based on the exclusion criteria described in the materials and methods section, two sets of 34 possible unique patient–relative pairs were generated for both the serum and stool samples. These two datasets of patient-relative pairs were used to evaluate whether the specific pairs chosen influenced certain metabolites that were found to be significant contributors to the discrimination between FD patients and healthy controls. 

Both lists (paired version 1 and 2) of FD patient-relative pairs yielded comparable results when subjected to a paired volcano analysis (FC ≥ 1.2, FDR corrected *p* value < 0.05), identifying two serum metabolites, urea and xanthine, as significant discriminators between FD patient and healthy relative pairs (Table 2). Similar paired analyses were conducted with stool metabolite profiles, which identified three metabolites, choline, malonate, and taurine, as significant discriminators of an FD patient from their respective healthy control relative. All three stool metabolites were in higher concentrations in FD patients’ samples compared to their paired relatives’ samples (Table 2). 

Overall, the order and extent of significance (as assessed by FDR corrected *p* values) of the metabolites identified as discriminating between the paired subject groups were influenced by which list of patient-healthy relative pairs was chosen for analysis. However, similar metabolites were found to be important, irrespective of which paired list was used. In the case of the population-wise (unpaired) analysis, all patients and relatives were represented with a single biological replicate, thus allowing for the identification of a greater number of significant metabolites. Paired ratios have only a subset of the samples, which likely does not provide sufficient statistical power to attribute some of the metabolites as significant in the paired analysis compared to the unpaired analysis.

Considering results from all these analyses and a thorough metabolic pathway review, serum metabolites that were distinct contributors to the separation of the FD patient group from their healthy control relative group, and that alluded to potentially impacted metabolic pathways, could be classified as markers of energy homeostasis pathways (amino acid catabolism, branched chain amino acids (BCAAs), and urea), tricarboxylic acid (TCA) cycle activity and mitochondrial function (taurine), and the purine salvage pathway (xanthine and inosine). Stool metabolites that were consistent discriminators of FD patients from healthy relative controls and connected to distinct metabolic pathways could be grouped as neuronal metabolites (choline, taurine, beta-alanine, and tyramine) and gut-microbial related metabolites (choline, p-cresol, tyramine, beta-alanine, and valerate). 

## 4. Discussion

Our extensive analysis of polar serum and stool metabolite profiles indicated distinct metabolic shifts in FD patients compared to what is observed in healthy control relatives. Due to the rarity of FD, this study achieved a statistically rich sampling of the population of individuals afflicted; herein, we analyzed ~14% of all individuals diagnosed with FD worldwide. Both serum and stool polar metabolite profiles provided snapshots of the systemic and gut/excretory metabolic states of FD patients and resulted in a clear separation of FD patients from healthy relative controls based on distinct metabolic characteristics. These findings are particularly remarkable given that the serum and stool samples were collected from FD patients in uncontrolled environments exhibiting diverse phenotypic traits and different degrees of disease severity. 

This analysis extends from work that we recently reported on the gut–brain–metabolism axis in FD [4], providing additional information on the serum and fecal metabolome changes observed in FD patients and their potential significance. The present study analyzes metabolome data by population (FD patients versus control group) and in a pairwise fashion (each FD patient matched to their cohabitating healthy relative control), rather than exclusively using a permutation algorithm of pairwise samples [4]. Comparing results from the population and pairwise analyses aimed to minimize the influence of confounding factors among patients or relatives, which could have included the environment (patients and matched relatives likely share distinct environments); genetics (parents of FD patients have one copy of the *ELP1* gene mutation and are a close genetic match); and diet (cohabitating individuals likely share comparable diets, when possible).

In our previous analysis [4] of microbiome and metabolome changes, additional variables such as sex, weight, body mass index, age, use of antibiotics, fundoplication (a gastric surgery to reduce acid reflux), and use of a gastrostomy tube (G-tube) were all assessed for potential correlations to the changes observed. The only correlations between the metabolite levels and these external parameters involved patients who had received fundoplication surgery and stool choline levels. However, out of 41 patients who donated stool samples, 28 had received fundoplication surgery, representing a larger group than patients without (*n* = 13). Thus, our results on stool choline levels are unlikely to have been skewed by this association. 

In order to prevent adverse health effects from our sampling effort on the patient population, we did not request that stool and serum samples be collected at specific times or restrict patients to the same diet as their control relatives. Patients were permitted flexibility regarding when and where they collected and provided samples, which may have resulted in some of the heterogeneity in the metabolite patterns observed and could subsequently mask the significance of certain metabolite differences. The population and pairwise analyses were undertaken to ensure that metabolites of interest, which are potentially relevant to FD, would not be overlooked by solely relying on a random selection approach of patient-relative pairs ([4]), while still maintaining an unbiased approach to interrogate the data. Results from the current more detailed analysis of FD patient metabolite profiles clearly demonstrate that the mutation in the *ELP1* gene responsible for FD is associated with multiple notable changes in patient metabolomes, and provides strong support to the physiological data that suggests FD patients experience significant metabolic deficits [32,33].

Of the 55 serum and 73 stool polar metabolites identified and quantified, differences in the levels of eight serum and eight stool metabolites were found to be of significant interest through both unpaired and paired statistical analyses and thorough pathway impact analyses. For serum samples, these metabolites included urea, which was found to be significantly elevated in the FD patient group (Supplementary Table S2). In contrast, the serum metabolites xanthine, inosine, leucine, valine, taurine, isoleucine, and alanine were either significantly decreased (as assessed by statistical FDR corrected *p* values) or trending toward being in lower concentrations in the sera of FD patients compared to the levels measured in the sera of the healthy control relative group (Supplementary Table S2). 

With respect to stool samples, the six metabolites (beta-alanine, choline, malonate, tyramine, taurine, and p-cresol) were found to be significantly elevated in the FD patient group compared to the healthy relative controls. In contrast, levels of fumarate and valerate trended toward being lower in FD patients (Supplementary Table S3). 

**Figure 3:**
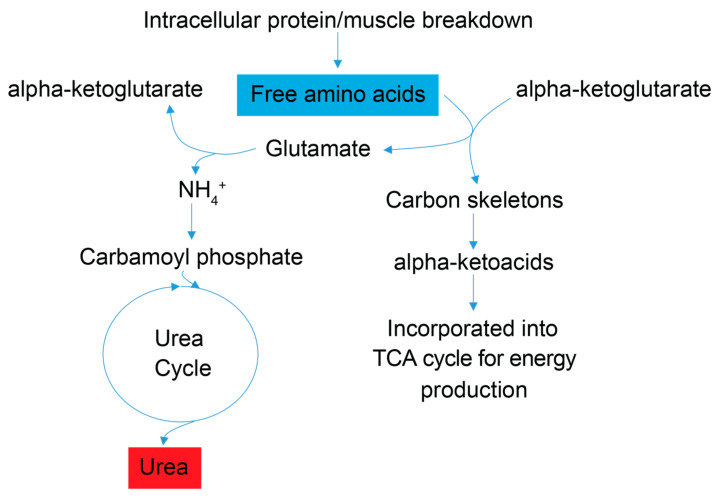
Simplified schematic of amino acid catabolic pathways used for ATP production. Enhanced amino acid degradation, a signature of energetic needs, is supported by an increased concentration of urea in FD patient sera. As indicated by enhanced catabolism of the amino acids alanine and asparagine, and the BCAAs valine, leucine and isoleucine, all trending to be lower (blue). Subsequently, a byproduct of the catabolism of amino acids for energy homeostasis is urea (red), which is then excreted. Red metabolites are increased in FD patient sera, and blue metabolites are decreased.

**Figure 4:**
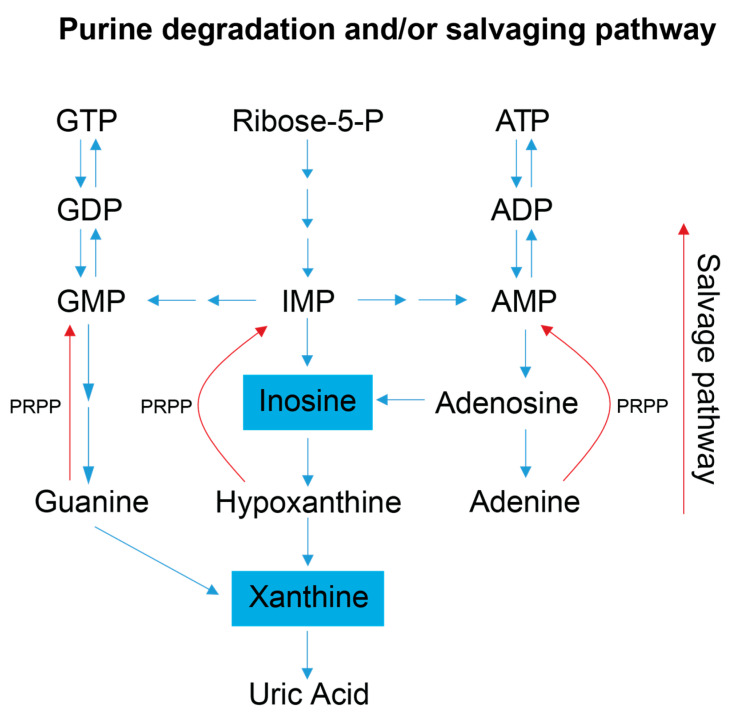
Simplified schematic of the purine salvage pathway. This diagram highlights how a decrease in purine degradation intermediates could reflect the salvaging of purines to preserve energy. Inosine is produced as a result of adenosine deamination via the action of adenosine deaminase and is a key intermediate of purine catabolism in humans, whereas xanthine is generated by the action of xanthine oxidase and is the product of oxidation of hypoxanthine (created via the conversion of inosine) and concomitant reduction of NAD+ to NADH. Additionally, xanthine can be produced via the deamination of guanosine. Xanthine oxidase catalyzes the oxidation of xanthine to uric acid, which is normally excreted in the urine, and was not detected in our metabolite profiling analysis of the serum samples [1,2]. Blue colored metabolites indicate decreased concentration in FD patient sera. Abbreviations: PRPP, phosphoribosyl pyrophosphate; IMP, inosine monophosphate; AMP, adenosine monophosphate; ADP, adenosine diphosphate; ATP, adenosine triphosphate; GMP, guanosine monophosphate; GDP, guanosine diphosphate; GTP, guanosine triphosphate.

Examination of the serum and stool metabolite patterns measured in FD patients support previous clinical reports suggesting FD patients experience metabolic energy deficits [7]. In addition, this NMR-based metabolomics study identified other metabolic pathways that are likely implicated in the phenotypic expression of FD. The clinical premise that FD patients suffer from a metabolic energy deficit is supported by our observations that the levels of several serum metabolites associated with energy (ATP) production and nucleotide salvage pathways are altered in FD patients compared to controls. These include metabolites associated with amino acid catabolism (alanine, BCAAs, and urea), the purine salvage pathway (xanthine and inosine), and mitochondrial dysfunction (taurine) (Figure 3 and Figure 4).

**Figure 5:**
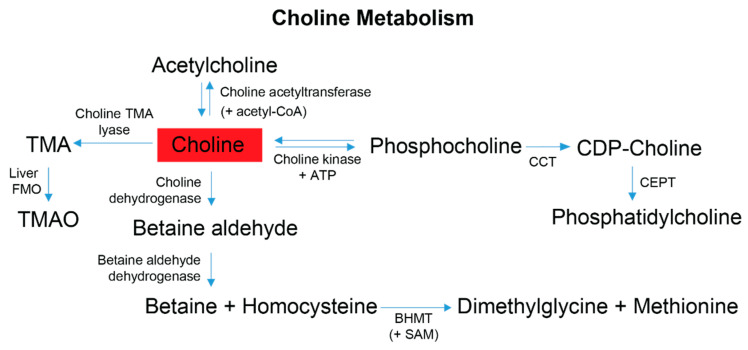
Choline is obtained primarily from the diet and contributes to four metabolic pathways employed by gut microbes and the host. Dietary choline is preferentially committed to the Kennedy pathway to produce phosphatidylcholine; this process is a 3-step reaction requiring initial ATP expenditure [75]. At the synaptic cleft, choline can also undergo a cyclical reaction to produce acetylcholine for autonomic signaling transduction [66,76,77]. Within the liver and kidneys, choline can be converted to betaine, which is an osmotically active compound and a primary methyl donor that helps facilitate the re-methylation of homocysteine to methionine [66,68]. The production of TMA is mediated by the gut-microbiome. Following gut microbial conversion, TMA is classically absorbed by the gut epithelium, transported to the liver and converted by Flavin-monoxygenases (FMOs) to trimethylamine-N-oxide (TMAO) [51,66]. Red indicates choline was elevated in the stool metabolome of FD patients compared to their healthy relatives. Abbreviations: CoA, coenzyme-A; ATP, adenosine triphosphate; CPT, choline-phosphate cytidyltransferase; CDP, cytidyl-diphosphocholine; CEPT, choline ethanolamine phosphotransferase; BHMT, betaine homocysteine methyl transferase; SAM, S-adenosyl methionine; TMA, trimethylamine; TMAO, trimethylamine-N-oxide.

A metabolic pathway impact analysis was undertaken for the stool metabolome data using literature information and MetaboAnalyst v.4.0. Such analyses resulted in the grouping of the eight metabolites of interest in four different categories as follows: (1) choline and taurine; (2) tyrosine, fumarate, and p-cresol; (3) beta-alanine and malonate; and (4) valerate (Figure 5 and Supplementary Figures S9–S11). Of these metabolites and pathways impacted, those with relevance to the gut microbiome and nervous system were of primary interest and presented potential insights into how metabolic networks impact FD disease traits through gut-microbe dysbiosis and/or exacerbation of neurodegeneration. Four of the eight stool metabolites identified as being significant and relevant to neural signaling included choline, taurine, beta-alanine, and tyramine (Supplementary Figure S12). Five of these eight metabolites could also be associated with gut microbes, and included tyramine, p-cresol, valerate, beta-alanine and choline. The gut-microbial and metabolic conclusions drawn for these metabolites regarding FD are consistent with published reports highlighting mechanisms by which the gut microbiome contributes to gut health and functionality, disease development, energy homeostasis, bioactive compound production, and the modulation of the communication within the gut–brain axis [1,2,3,34,35,36,37].

The distinct serum and stool metabolic profiles of FD patients support clinical observations and revealed metabolic changes in FD disease at the systemic and gut-function levels (discussed further below). Together, these data support the notion that disruption in nutrient absorption and gut microbiome metabolic efficacy contribute to an overall systemic energy deficit, and lead to compensatory metabolic adaptations giving rise to altered polar metabolite patterns, as observed in this study. Lastly, taurine exhibited interesting trends in both serum and stool samples (trending toward a decrease in serum and an increase in stool), suggesting a possible systemic deficit of taurine induced by either diet or gut-absorption capacity with potentially profound impacts on host metabolism.

### 4.1. Amino Acid Catabolism Suggests an Overall Energy Deficit

Signatures of energy deficits are reflected in the sera of FD patients by observations of lower levels of the amino acid alanine and the branched chain amino acids (BCAAs) valine, leucine, and isoleucine, which suggest increased amino acid catabolism; and by elevated urea concentration, which points to increased nitrogen excretion and is consistent with increased amino acid degradation in FD patients. Degradation of skeletal muscle protein is enhanced under starvation/energy deficient physiological states, and an increase in BCAA catabolism is commonly associated with starvation and muscle wasting, including the debilitating muscle wasting disease rhabdomyolysis [38,39], which has been shown to increase systemic urea levels and can harm kidney function [40]. FD patients are prone to low muscle mass and some suffer from rhabdomyolysis [32]. Blood tests for glomerular filtration rate, blood urea nitrogen content, and creatinine levels (Supplementary Figure S1) indicated that only two FD patients who provided samples displayed signatures of kidney dysfunction. Higher serum urea levels in FD patients, thus, reflect, in all likelihood, increased the deamination of amino acids and the excretion of excess nitrogen associated with amino acid catabolism, while amino acid carbon scaffolds are directed to ATP generating pathways (Figure 3). Enhanced amino acid catabolism is also a compensatory response to mitochondrial dysfunction (providing amino acid precursors for de novo glucose synthesis and the subsequent use of glucose in glycolysis), impaired oxidative phosphorylation and aerobic ATP energy production, which have been reported to be hallmarks of FD and other neurological diseases including Alzheimer’s and Parkinson’s [14,16].

### 4.2. Purine Salvage and Nucleotide Degradation Pathways

FD patient sera have significantly lower xanthine levels, and trend toward lower serum inosine levels, suggesting increased purine salvaging compared to healthy relative controls (Figure 4) [41,42]. Because de novo synthesis of purine nucleotides is an energy expensive process [43,44], decreased levels of the intermediates involved in purine degradation suggest that FD patients may be minimizing the excretion of purine bases and maximizing the purine salvage pathway to minimize the energetic demands of de novo purine biosynthesis. FD patients are known to undergo hypoxic stress, especially during sleep, and many die due to sudden unexpected death during sleep (SUDS) [45]. Since purine nucleotides (i.e., adenosine) are also salvaged and utilized to cope with hypoxic and oxidative stress, it is reasonable to postulate that this process may also be occurring in FD patients [46]. Decreased purine degradation and enhanced purine salvaging are also observed in other neurological diseases that share hallmark clinical features with FD including AD, PD, ALS, and ASD [47,48,49]. 

### 4.3. Tyrosine Degradation

The tyrosine degradation pathway combines reactions that catalyze the production of tyramine, p-cresol, and fumarate (Supplementary Figure S9). The production of tyramine and p-cresol, both of which are higher in concentration in stool samples of FD patients, are solely dependent on microbial activity in food and the gut [50,51,52,53]. Conversely, the production of fumarate, which is lower in concentration in stool samples of FD patients, links to the TCA cycle and the electron transport chain (ETC) of the host and gut-microbes. However, the reduced energetic status of the FD patients, as discussed in Section 4.1 and Section 4.2, suggest limited TCA cycle activity, thereby limiting the production of fumarate from this cycle. Bacterial fermentation leads to the production of fumarate as an intermediate, which has low bioavailability due to its extensive utilization by gut bacteria [54]. Changes in fumarate levels in the stool may, thus, reflect altered bacterial activity or the presence of specific microbes, as reported in our previous paper [4]. 

### 4.4. Metabolites Implicated in Gut Microbiome Changes

The criteria used in our analyses to establish the association of specific stool metabolites with the gut microbiome were as follows: (1) The metabolites of interest were reported in the literature to be produced or utilized by a genus or species of bacteria; or (2) had been correlated with promoting or impairing the growth of a given genus in published studies; or (3) their presence has been associated with the abundance of a specific microbial genus or species. Interestingly, all five of the metabolites classified as being associated with gut microbes (tyramine, p-cresol, valerate, beta-alanine and choline) were found to be associated with *Clostridium* in other studies [36,37,50,55,56,57,58]. Elevated levels of beta-alanine, tyramine and p-cresol were positively correlated with an increase in *Clostridium* genera. Conversely, the gut protective short chain fatty acid (SCFA) valerate has been shown to inhibit the growth of *C. difficile* in vitro [59]. Interestingly, FD patients exhibit elevated levels of tyramine, p-cresol, and beta-alanine in their stool samples, with decreased levels of valerate, findings that are consistent with FD patients being prone to develop *C. difficile* infections [7]. 

The association between *C. difficile* and p-cresol production is a specific field of research [60,61,62], in addition to the relevance of p-cresol to Autism Spectrum Disorder (ASD) which has been found to be elevated in stool samples of ASD patients [63,64,65]. FD patients are prone to *C. difficile* infections [7] and exhibit similar hallmarks to ASD regarding developmental impairments [5,66] and Elongator subunit protein dysfunction [13,18]. This suggests that p-cresol could be a valuable marker of disease that warrants further investigation. Additionally, excess p-cresol has been shown to cause DNA damage in vitro and to compromise colonic epithelial cell integrity [62,63], phenotypes that have been observed in FD mouse models and which are suspected to occur in FD patients as well [10,67]. Though additional gut microbes produce p-cresol and cannot be entirely ruled out for the changes in p-cresol levels observed in this study, the literature regarding p-cresol and *C. difficile* does suggest an interesting link between the susceptibility of FD patients to *C. difficile* infections and the associated downstream effects of p-cresol.

Valerate is a five-carbon SCFA which is produced by a select group of gut microorganisms [68,69]. Similar to the more common SCFAs (acetate, propionate, and butyrate [34]), valerate exhibits strong gastrointestinal (GI) protective effects, stimulates the growth of intestinal epithelium [36,59], and modulates autoimmune inflammation in the CNS [68]. As with other SCFAs, valerate is relevant to neurological and GI diseases that share hallmarks with FD [59,65,70]. Elevated levels of fecal SCFAs could be due to elevated production of these compounds by the gut microbiome, a reduction in epithelial absorption capacity, or a combination of both [71]. Valerate and other SCFAs present an interesting group of metabolites for further investigations, because they display synergistic relationships with the host metabolism, gut protective effects and, to a lesser extent, are linked to the production of malonate as an intermediate of SCFA biosynthesis [72]. 

An additional source of malonate in the gut might originate from microbiome-derived beta-alanine metabolism. Beta-alanine is produced from aspartate and uracil, and can be metabolized into malonate and carnosine (Supplementary Figure S10). Muscle carnosine primarily functions as a proton buffer, enhances muscle endurance [73], and is relevant to FD because patients struggle to maintain adequate muscle mass. The elevation of fecal beta-alanine highlights another possible absorption deficit with consequential detrimental effects. However, due to the variety of sources of beta-alanine (dietary, host metabolism, and gut microbiome) and the limited studies on malonate’s relevance in fecal samples, the factors that lead to elevated stool beta-alanine and malonate levels in FD patients are not fully understood. 

Another metabolite with microbiome relevance is choline, which is obtained from the diet and has four metabolic fates, one of which is initiated by the microbiome [74] with the remaining three pathways subject to influence from the gut microbiome outcompeting the host for the bioavailability of choline [58]. Adequate dietary choline is necessary for phosphatidylcholine, acetylcholine and betaine synthesis (Figure 5), with phosphatidylcholine accounting for 95% of the total choline pool in tissues [75]. Various studies have shown that perturbations of host choline metabolism result in liver damage, elevated markers of DNA damage, kidney dysfunction, and a reduction in mitochondrial membrane potential [37,58,75,76,77,78,79], all symptoms experienced to different degrees by FD patients and/or FD mouse models [5,9,80,81,82]. Partnering these symptoms with the observation of elevated choline levels in FD patient stool samples, and results reported in [4], suggests that host and/or gut microbial choline metabolism and/or choline absorption is altered in FD.

Though an unambiguous conclusion cannot be made regarding the microbial species’ origins of the observed stool metabolite patterns, an inference about gut health can be drawn, as the published studies cited above present important examples of severe gut dysbiosis and include the same metabolites that were identified in FD patients. These findings further substantiate the hypothesis that FD patients experience gut microbiome dysbiosis, which links to impairment of metabolic homeostasis. Our experimental results, which are based on detailed polar metabolite profile analyses, are consistent with microbiome findings that we reported in an earlier publication [4]. Patient antibiotic use was assessed in our previous publication and an association with the use of antibiotics and microbiome diversity was observed. However, no significant correlations with metabolite concentrations were found. The FD mouse study reported in our earlier publication also suggested that neuronal *Elp1* deletion was sufficient to drive microbiome diversity and stool metabolome separation [4]. Therefore, we suspect that the use of antibiotics may influence the microbiome, but from our initial assessment of metabolite patterns and our FD mice results, it does not seem to be a main variable driving the separation observed in the metabolome of FD patients compared to their healthy relatives.

### 4.5. Neuronal Associated Metabolites

Four metabolites (choline, taurine, beta-alanine, and tyramine) associated with neuronal health, and identified in this study, either contribute directly to nervous system signaling or are precursors of neurotransmitter biosynthesis (Supplementary Figure S12). Choline plays a crucial role in the nervous system through its involvement in the direct production of acetylcholine, a major neurotransmitter (Figure 5 and Supplementary Figure S12), the synthesis of sphingomyelin from phosphatidylcholine for myelination [77,83,84], and choline storage and membrane sustainability through phosphatidylcholine synthesis. Cholinergic parasympathetic neurons modulate function of the GI tract, a process that appears to be greatly impacted in FD patients, as noted by severe gastroenteropathy, abnormal GI tract coordination, and reduced innervation [7,80]. Sphingomyelin is a primary constituent of myelin, which is reduced in large and small sensory axonal fibers in FD patients [5]. Cholinergic neurons are particularly vulnerable to altered choline metabolism, as choline is used both as a membrane lipid for structure and choline storage, and as the neurotransmitter acetylcholine. Given this dependence on choline, failure in its absorption may also contribute to the death of cholinergic neurons in FD based on altered choline metabolism [85]. The production and utilization of choline as acetylcholine is a cyclic process; therefore, a loss of choline in overactive metabolically taxed neurons depletes cellular membrane stores of phosphatidylcholine, further impairing cell functions and promoting neurodegeneration [37,86].

The gamma-aminobutyric acid-a (GABAa) receptor gene is downregulated in stem cells derived from FD patients [87], and GABAa receptor agonists have been used to treat FD crises experienced by patients [7]. Taurine and beta-alanine both bind GABA receptors [88] and beta-alanine can regulate both the GABA [89] and taurine transport systems [90], which reduce cellular taurine transport, preventing the ability of taurine to improve calcium transport, and to modulate protein phosphorylation and regulate oxidative stress (see Section 4.6) [91].

Lastly, tyramine binds to baroreceptors to increase blood pressure. It has been recommended that FD patients restrict dietary tyramine consumption to help prevent hypertensive crises [92]. Tyramine present in foods and produced by the gut is metabolized by Monoamine Oxidase (MAO) A in the GI tract. However, tyramine is not degraded in individuals taking MAO inhibitors or in patients, including FD patients, who are deficient in MAO [92,93]; this thus allows excess tyramine to enter the systemic circulation through epithelial absorption [94] and ultimately triggers hypertensive crises, through adrenergic norepinephrine production, which are experienced by FD patients [5,93,94].

### 4.6. Taurine Metabolism and a Potential Dietary Deficit with Systemic Consequences

Taurine is of significant interest as its concentration is both elevated in the stool and lower in the sera of FD patients, suggesting a possible systemic deficit of this crucial metabolite. Taurine is produced endogenously via the degradation of homocysteine (Supplementary Figure S11), is one of the most abundant amino acids in mammals [95], and, similar to choline, is not only produced endogenously but obtained in substantial quantities from the diet. The elevated and decreased levels of taurine observed in the FD patients’ stool and serum samples, respectively, could thus reflect an absorption impairment. In addition to the function of taurine in the nervous system (discussed above), another established biological fate of taurine is to enter the hepatic recycling loop via conjugation with bile acids (Supplementary Figure S11). The conjugation of taurine to bile acids enhances their solubility and improves the dietary absorption of lipids and lipid soluble vitamins [96,97]. Following facilitated absorption, bile acids are deconjugated by gut microbe activity, reabsorbed, and recycled. If FD patients do indeed exhibit impaired bile acid deconjugation resulting from microbiome dysbiosis, reduced cycling of taurine-conjugated bile acids could exacerbate GI dysfunction and further promote systemic deficits of taurine and other dietary nutrients.

Taurine deficient phenotypes are characterized by retinal degeneration, cardiomyopathy, and skeletal muscle malfunction [90], as a result of mitochondrial deficits including reduced complex I ETC activity, TCA activity and oxygen consumption and increased ROS production [98]. Mitochondrial dysfunction has been demonstrated in FD mouse models [10,11,67] and observed in an FD patient muscle biopsy which revealed a functional impairment of mitochondrial complexes I, III and IV, which are integral protein components of the ETC [10]. ETC impairment has also been reported to contribute to disease progression in AD, PD, and ASD [14,16,99,100,101]. Interestingly, characteristics of taurine deficiency are also observed in FD patients [5,32] and FD mouse models [10,67]. Being the most abundant amino acid in the retina, and the fact that taurine deficits cause degeneration of retinal ganglion cells (RGC) [102,103,104], i.e., the same cell type that dies progressively in FD patients [10,67], taurine levels may be relevant to the progressive blindness experienced by FD patients as they age.

## 5. Conclusions

In conclusion, our NMR-based metabolomics analyses demonstrated that FD patients exhibit significant changes in their stool and serum polar metabolomes. These metabolic alterations indicate the importance of the gut–brain–metabolism axis in FD, and support recently published microbiome findings [4]. Knowledge gained from these studies in FD may be applicable to other neurodegenerative diseases including AD and PD, which share disease hallmarks with FD [13,14,15,16]. Results from our serum polar metabolite profile analyses demonstrated that FD patients present with energetic (ATP) deficiencies, and that metabolic pathways associated with energy generation or metabolite sparing are impacted. From analysis of the serum metabolomics data, we found that the metabolic pathways impacted include enhanced amino acid catabolism, and the purine salvage pathway. Stool metabolome analyses have identified metabolites associated with pathways involved in neuronal signaling, and indicators of gut-microbiome health and function. Metabolic patterns observed in the profiles of FD patients are consistent with reports from other neurological diseases including PD, AD, ALS, and ASD, suggesting that metabolic impairments observed in FD apply to and/or are relevant to other neurological diseases. New knowledge gained about the mechanisms associated with altered metabolism in FD has the potential to aid in understanding other, more complex, and multifactorial neurological diseases where the gut–brain–metabolism axis plays a prominent role. Knowledge about the key molecular indicators of disease may help guide the design of intervention therapies which, by restoring metabolic homeostasis and/or a healthy gut microbiome, could mitigate disease symptoms, and redress or slow down neurodegeneration.

## Figures and Tables

**Table 1:** Unpaired volcano plot analysis of serum and stool metabolite concentrations between the FD patient and control relative sample groups.

**Serum Metabolites**	**Fold Change (FC)**	**log_2_(FC)**	***p* Value (FDR)**	**−LOG_10_(p)**
Xanthine	0.53	−0.90	1.90 × 10^−4^	3.72
Urea	1.44	0.53	1.69 × 10^−3^	2.77
π-Methylhistidine	0.59	−0.75	1.44 × 10^−2^	1.84
1,7-Dimethylxanthine	0.55	−0.86	3.80 × 10^−2^	1.42
Dimethyl sulfone	0.72	−0.47	3.80 × 10^−2^	1.42
Methanol	0.73	−0.46	3.80 × 10^−2^	1.42
**Stool Metabolites**	**Fold Change (FC)**	**log_2_(FC)**	***p* Value (FDR)**	**−LOG_10_(p)**
Choline	3.41	1.77	8.22 × 10^−6^	5.08
Malonate	3.17	1.66	3.15 × 10^−3^	2.50
β-Alanine	5.42	2.44	0.26	0.58
Tyramine	1.93	0.95	0.26	0.58
p-Cresol	1.42	0.51	0.28	0.54

Unpaired volcano plot analysis indicating fold change (FC) and two-sample *t*-test significance. A fold change threshold of ≥1.2 and a *p* value with FDR correction < 0.05 (serum) and <0.3 (stool) were set as significance criteria. Fold changes were taken as the ratio of the mean metabolite concentrations for the FD patients’ group compared to the healthy control relatives’ group prior to the application of statistical normalization (log transformation and autoscaling).

**Table 2:** Paired volcano analysis of serum and stool metabolite profiles obtained from the v.1 and v.2 list of FD patient–relative control pairs.

**Serum Paired Volcano Analysis Version 1:**				
	**FC**	**log_2_(FC)**	***p* Value (FDR)**	**−LOG_10_(p)**
Xanthine	0.40	−1.34	1.44 × 10^−2^	1.84
Urea	1.38	0.47	1.44 × 10^−2^	1.84
**Serum paired volcano analysis version 2:**				
	**FC**	**log_2_(FC)**	***p* value (FDR)**	**−LOG_10_(p)**
Xanthine	0.35	−1.51	3.26 × 10^−3^	2.49
Urea	1.39	0.48	1.15 × 10^−2^	1.94
**Stool paired volcano analysis version 1:**				
	**FC**	**log_2_(FC)**	***p* value (FDR)**	**−LOG_10_(p)**
Choline	3.36	1.75	1.19 × 10^−3^	2.92
**Stool paired volcano analysis version 2:**				
	**FC**	**log_2_(FC)**	***p* value (FDR)**	**−LOG_10_(p)**
Choline	2.34	1.23	3.58 × 10^−2^	1.45
Malonate	2.14	1.10	6.69 × 10^−2^	1.17
Taurine	3.04	1.60	8.65 × 10^−2^	1.06

Two separate, randomly generated paired datasets for both serum and stool metabolite data. A paired volcano analysis was performed for each paired dataset. Fold change (FC), logarithmic fold change (log_2_(FC)), and paired *t*-test *p* values are presented. The fold change threshold was ≥1.2 and ≤0.8, and the FDR corrected *p* value threshold was <0.05 (serum) and <0.1 (stool). Fold change values for each metabolite were obtained from average values measured in FD patients/Control Relative pair ratios; a negative FC indicates lower levels in FD patients compared to their paired relatives, whereas a positive FC indicates higher levels in FD patients.

## Data Availability

The data presented in this study are openly available in MetaboLights at https://www.ebi.ac.uk/metabolights/MTBLS5138/descriptors (accessed on 6 December 2022), reference number MTBLS5138.

## Supplementary Materials

The following supporting information can be downloaded at: https://www.mdpi.com/article/10.3390/metabo13030433/s1. Table S1: Summary of clinical data from de-identified FD patients and control relatives. Table S2: Polar metabolites identified and quantified in the serum samples of FD patients and healthy relative controls. Table S3: Polar metabolites identified and quantified in the stool samples of FD patients and healthy relative controls. Table S4: Variable Importance in Projection (VIP) scores associated with components 1, 2 and 3 of the PLS-DA model generated from the metabolomics analysis of the serum samples of FD patients and healthy control relatives. Table S5: Variable Importance in Projection (VIP) scores associated with components 1, 2 and 3 of the PLS-DA model generated from the metabolomics analysis of the fecal samples of FD patients and healthy control relatives. Figure S1: Results from clinical blood tests of de-identified FD patients. Figure S2: Results from liver enzyme blood tests and measurements of ALT and AST levels in de-identified blood samples of FD patients. Figure S3: Characteristic 1D 1H NMR spectrum recorded on serum metabolite extracts obtained from FD patients and healthy relative controls. Figure S4: Characteristic 1D 1H NMR spectrum recorded on stool metabolite extracts obtained from FD patients and healthy relative controls. Figure S5: Validation metrics of the PLS-DA modeling of serum metabolite profiles. Figure S6: Validation metrics of the PLS-DA modeling of stool metabolite profiles. Figure S7: Scree plot of the PCA modeling of both serum and stool metabolite profiles. Figure S8: Random forest analysis of the serum and stool metabolite profiles of the FD patient and healthy control relative groups. Figure S9: Schematic diagram of metabolic pathways involved in tyrosine catabolism. Figure S10: Schematic diagram of metabolic pathways involved in beta-alanine and malonate metabolism. Figure S11: Schematic diagram of metabolic pathways involved in taurine metabolism. Figure S12: Schematic diagram of four significant metabolites identified in the stool of FD patients and identified as important neuronal cell signaling molecules. Refs [74,85,89,91,95,105,106,107] are citied in the Supplementat Materials.
