# Supplementary material for: A Comprehensive NMR Analysis of Serum and Fecal Metabolites in Familial Dysautonomia Patients Reveals Significant Metabolic Perturbations
Source: Metabolites. 2023 Mar 16;13(3):433. doi: 10.3390/metabo13030433 (PMC10057143; doi:10.3390/metabo13030433)
Supplement: Supplementary file 1 [file metabolites-13-00433-s001.zip › metabolites-2235063-supplementary.pdf]

Supplementary Information:

Title: A comprehensive NMR analysis of serum and fecal metabolites in Familial dysautonomia patients reveals significant metabolic perturbations

Authors: Stephanann M. Costello[†], Alexandra M. Cheney[†], Annie Waldum, Brian Tripet, Maria Cotrina-Vidal, Horacio Kaufmann, Lucy Norcliffe-Kaufmann, Frances Lefcort, and Valérie Copié*

Costello, Cheney et al., Supplementary Table S1

Table S1. Patient and control relative sample and clinical data summary.

**Patient Data:** **one patient declined to provide info*	**Number**	**Percent**	**Control Relative Data:**	**Number**	**Percent**
Total number of patients enrolled:	50	100%	Total number of control relatives enrolled:	54	100%
Patients only providing stool samples:	7	14%	Controls only providing stool samples:	7	13%
Patients only providing serum samples:	9	18%	Controls only providing serum samples:	8	15%
Patients providing both serum and stool:	34	68%	Controls providing both serum and stool:	39	72%
Total number of patients providing stool:	41	82%	Total number of controls providing stool:	46	85%
Provided second biological stool replicate:	12	24%	Provided second biological stool replicate:	11	20%
Provided third biological stool replicate:	2	4%	Provided third biological stool replicate:	1	2%
Total number of stool samples provided:	54		Total number of stool samples provided:	58	
Total number of patients providing serum:	43	86%	Total number of controls providing serum:	47	87%
Provided second biological serum replicate:	6	12%	Provided second biological serum replicate:	6	11%
Total number of serum samples provided:	49		Total number of serum samples provided:	53	
Female patients	29	58%	Female controls	30	56%
Male Patients	21	42%	Male controls	24	44%
Age group <12 years	8	16%			
Age group 12-17 years	5	10%			
Age group 18-24 years	16	32%			
Age group 25-34 years	16	32%			
Age group 35-44 years	3	6%			
Age group above 45 years	1	2%			
Underweight BMI <18.5	20	40%			
Healthy BMI 18.5-24.9	27	54%			
Overweight BMI 25.0-29.9	2	4%			
Has a gastric tube:	40	80%			
Had a fundoplication:	36	72%			
Can consume food orally:	37	74%			
Can consume liquid orally:	17	34%			
Can consume mixed foods orally:	10	20%			
Can only use gastric tube for consumption:	11	22%			
Received Antibiotics 3 months prior to sample donation	20	40%	Received Antibiotics 3 months prior to sample donation	2	4%

Stool and serum sample collection data for FD patients and control relatives. Patient clinical data was also collected by the Familial Dysautonomia Center at time of collection of samples. Control relatives did not provide clinical data, but it is assumed the age gap is large due to relatives being the parent of the patients. One enrolled patient declined to provide clinical data. The percentage represents the percent out of those enrolled in the study in each group.

Costello, Cheney et al., Supplementary Table S2

Table S2. Human Serum Metabolites Identified and Quantified by NMR: Concentrations and Percent Differences

Metabolites	FD Patients (nmoles/mL)		Control Relatives (nmoles/mL)		Percent Difference
	Mean	Stdev	Mean	Stdev	Mean
1,7-Dimethylxanthine	4.7	4	8.5	10.3	-45
2-Hydroxybutyrate	24.8	18.4	31.8	25.1	-22
2-Oxoglutarate	2.9	1.1	3.1	1.4	-6
2-Oxoisocaproate	19.4	6.7	19.5	6.9	-1
3-Hydroxybutyrate	89.2	99	91.6	115.2	-3
3-Hydroxyisobutyrate	19.6	8	19.2	5.8	2
3-Methyl-2-oxovalerate	18.8	5.5	19.2	6.3	-2
Acetate	79.4	30.4	76.3	52.5	4
Acetone	18.6	11.9	18.3	12.4	2
Alanine	221.2	61.7	247.3	65	-11
Arginine	55.8	19.5	56.4	21.1	-1
Asparagine	27.2	7.2	24.9	6.3	9
Betaine	61.8	30.5	53.2	19.7	16
Butyrate	29.5	14.8	30.2	11.3	-2
Cadaverine	19.2	6.5	20.8	8.5	-8
Capric acid	1315.2	607.3	1446.8	590.2	-9
Carnitine	40.2	10.8	44	12.5	-9
Choline	18.1	12.4	17.3	10.8	5
Creatine	26.8	24.7	25.9	14	3
Creatinine	84.4	73.1	78.4	19.2	8
Dimethyl sulfone	5.2	3	7.2	5.7	-28
Dimethylamine	1	0.6	1.2	0.7	-17
Formate	59.6	27.1	59	29.1	1
Glucose	4465.2	847.4	4380.5	909.6	2
Glutamine	295.4	68.3	271.8	56.3	9
Glycerol	74.6	25.9	65.8	28.7	13
Glycine	167.4	75	136.2	54.9	23
Glycolate	23.4	12.6	34.5	20.4	-32
Histidine	41.7	11.9	40.2	11	4
Hypoxanthine	5.3	2	5.6	2.2	-5
Inosine	4.2	3.1	5.2	3.3	-19
Isobutyrate	7.7	3.4	7.5	3	3

Isoleucine	56.4	20.9	63.2	24.3	-11
Isopropanol	2.6	1.6	2.2	1.2	18
Lactate	1268.5	372.1	1271.3	330.8	0
Leucine	97.7	34.1	111.2	38.9	-12
Malonate	12.9	6.5	12.5	5.8	3
Methanol	14.2	12.2	19.5	11.9	-27
Methionine	19.7	7.4	19.3	5.2	2
O-Acetylcarnitine	3.5	1.6	3.4	1.8	3
O-Acetylcholine	3	0.9	3.1	1.4	-3
Phenylalanine	51.3	13.9	52.5	14.5	-2
Proline	350.1	150.2	342.7	123.5	2
Propylene glycol	122.6	100.1	93.6	27.8	31
Pyruvate	10.8	6	9.4	6.1	15
Serine	129.4	51	114.7	49.8	13
Succinate	7.4	3.8	7.4	4.1	0
Taurine	117	31.1	131.5	34.1	-11
Threonine	123.5	32.1	119.7	32.9	3
Tryptophan	69.5	29.2	74.8	22.2	-7
Tyrosine	56.9	25.7	61.1	18	-7
Urea	13987.3	7670	9718.3	2851.3	44
Valine	159.3	53.7	179.4	53.9	-11
Xanthine	8.6	11.9	16.1	12.6	-47
π-Methylhistidine	9.2	15.9	15.5	15.4	-41

Metabolite concentrations were normalized to serum volume and are reported in nmoles per mL. 55 metabolites were identified across 49 FD patient samples (from 43 patients total) and 53 control relative samples (from 47 control relatives total). Columns 1 and 2 report the mean metabolite concentrations measured in the sera of FD patients and healthy control relatives, respectively, and mean standard deviations. Column 3 reports estimates of percent differences (%) in mean metabolite concentrations between FD patients and healthy controls. A negative percent value indicates a mean lower in FD patients compared to controls, and a positive percent value is higher in FD patients compared to controls.

Costello, Cheney et al, Supplementary Table S3

Table S3. Stool Polar Metabolites Identified and Quantified by NMR: Concentrations and Percent Differences

Metabolites	FD Patients (nMol/g)		Control Relatives (nMol/g)		Percent Difference
	Mean	Stdev	Mean	Stdev	Mean
1,1-Dimethylbiguanide	104.2	263.3	2123.9	12595.8	-95
1,3-Dihydroxyacetone	111.3	149.3	118.5	94.8	-6
2′-Deoxyuridine	34.6	27.5	35.2	24.6	-2
2-Aminobutyrate	571.3	666.9	426.8	431.0	34
2-Aminoisobutyric acid	175.7	114.1	196.1	155.9	-10
4 Hydroxyphenylacetate	249.6	468.9	124.7	84.1	100
5-Aminopentanoate	2223.0	4868.0	987.4	1146.5	125
Acetate	66794.4	39628.5	60482.8	39076.2	10
Alanine	7547.0	6633.5	5945.0	3546.8	27
Arabinose	1379.1	1136.4	1574.2	2127.9	-12
Arginine	544.5	507.1	442.4	365.2	23
Aspartate	4012.5	2258.5	3469.9	1560.4	16
Butyrate	16309.6	11018.9	19333.8	18138.2	-16
Choline	310.3	431.7	91.0	60.8	241
D-Glucose	3207.5	3955.6	2527.8	3180.0	27
D-Mannose	226.3	463.0	259.1	451.9	-13
Dimethylamine	243.1	173.5	230.5	196.5	5
Dopamine	3813.8	25452.7	305.0	338.3	1150
Ethanol	28380.9	79317.3	8978.5	7565.3	216
Formate	2249.0	8536.6	274.4	289.3	720
Fumarate	310.7	624.2	310.7	478.6	0
Galactose	1227.3	715.7	1288.7	921.3	-5
Glucitol	2216.4	6656.0	1411.9	1404.0	57
Glutamate	8803.3	7109.1	7354.0	3990.8	20
Glutamine	1689.8	2220.4	1450.8	1244.8	17
Glutarate	691.9	805.9	936.7	2716.6	-26
Glycerol	4983.5	5504.4	6024.3	5134.1	-17
Glycerophosphocholine	730.1	1302.3	554.7	823.9	32
Glycine	4893.1	4846.3	3499.7	2361.6	40
Guanosine	42.5	64.2	43.0	64.0	-1
Histidine	543.1	887.1	354.2	238.4	53
Indole	265.4	202.6	190.3	95.3	40
Inosine	56.5	87.1	63.0	59.8	-10
Isobutyrate	2606.6	1326.4	2171.5	903.3	20
Isocaproate	566.0	780.2	394.5	193.6	44
Isoleucine	3891.9	3064.7	3159.6	1650.8	23
Isovalerate	2307.3	1472.6	1780.6	905.4	30
Lactate	6911.5	24769.0	648.0	593.6	967
Leucine	5429.8	3886.0	4383.7	2210.6	24

Lysine	5654.6	4223.0	4522.4	2287.1	25
Malic acid	1373.2	921.8	1585.4	962.4	-13
Malonate	3904.5	6925.3	1231.1	1521.4	217
Methionine	1965.9	1199.2	1828.1	980.5	8
Methylamine	382.1	267.5	439.6	379.5	-13
Methylsuccinate	164.4	144.8	222.2	222.5	-26
N-Acetylglutamic acid	394.4	244.5	371.2	221.7	6
Nicotinate	241.4	157.7	250.2	161.7	-4
Ornithine	1802.1	1697.4	1164.7	737.4	55
Orotic acid	458.3	538.1	628.8	886.3	-27
Phenylacetic acid	1726.5	948.9	1397.1	692.5	24
Phenylalanine	2085.4	1969.5	1639.3	916.4	27
Phosphoenolpyruvic acid	241.0	216.6	212.7	156.0	13
Proline	1977.4	2105.7	1620.4	1189.5	22
Propionate	13849.9	7215.2	13774.2	8087.9	1
Ribose	3643.0	2322.7	3818.4	2347.5	-5
Sarcosine	55.7	131.7	38.0	20.8	47
Succinate	7715.5	21266.8	2638.2	6086.3	193
Taurine	2334.7	3285.0	1287.0	2641.5	81
Threonine	2821.2	1853.8	2198.2	1129.8	28
Thymine	471.6	340.3	467.5	345.5	1
Trimethylamine	234.8	318.6	156.1	181.1	50
Tryptophan	576.3	553.3	405.2	227.5	42
Tyramine	182.2	317.8	94.3	57.4	93
Tyrosine	1885.4	1493.1	1565.9	803.4	20
Uracil	1198.3	1097.0	1264.7	909.8	-5
Uridine	137.1	157.1	146.2	126.6	-6
Urocanate	111.8	174.3	98.1	65.7	14
Valerate	2669.9	2655.8	2826.8	1702.0	-6
Valine	5171.5	4509.9	3870.5	2175.1	34
Xanthine	1597.2	1365.5	1877.2	1365.9	-15
Xylose	1426.9	1130.5	1165.6	888.7	22
p-Cresol	740.3	584.3	519.9	295.0	42
β-Alanine	1048.7	2290.6	193.6	309.4	442

Metabolite concentrations were normalized to dry stool mass and are reported in nmoles per gram. 73 metabolites were identified and quantified across 54 FD patient stool samples (from 40 patients total) and 58 control relative stool samples (from 46 control relatives total). Columns 1 and 2 report the mean metabolite concentrations measured in the stool of FD patients and healthy control relatives, respectively, and mean standard deviations. Column 3 reports estimates of percent differences (%) in mean metabolite concentrations between FD patients and healthy controls. A negative percent value indicates a mean lower in FD patients compared to controls, and a positive percent value is higher in FD patients compared to controls.

Costello, Cheney et al., Supplementary Table S4

Table S4. Variable Importance in Projection (VIP) scores associated with components 1, 2, and 3 of the PLS-DA model generated from the distinct serum metabolite profiles of the FD patients and healthy control relatives.

Metabolite	Comp. 1	Comp. 2	Comp. 3
Xanthine	2.80	2.64	2.44
Urea	2.45	2.41	2.18
pi-Methylhistidine	2.08	1.96	1.79
Dimethyl sulfone	1.86	1.76	1.64
1,7-Dimethylxanthine	1.79	1.71	1.65
Methanol	1.79	1.69	1.55
Glycine	1.47	1.39	1.40
Taurine	1.35	1.27	1.21
Alanine	1.31	1.23	1.39
Glycerol	1.22	1.15	1.06
Valine	1.21	1.19	1.23
Glutamine	1.15	1.09	1.09
Glycolate	1.13	1.09	1.03
Asparagine	1.11	1.28	1.16
Leucine	1.11	1.18	1.10
Inosine	1.03	0.99	0.93
Tyrosine	1.00	0.97	1.05
Isopropanol	0.98	0.95	1.08
Tryptophan	0.94	0.97	0.94
Serine	0.92	1.10	1.01
Carnitine	0.89	0.86	1.26
Isoleucine	0.85	0.95	0.95
Dimethylamine	0.82	0.81	1.07
Propylene glycol	0.82	0.78	0.77
Pyruvate	0.78	0.83	0.76
Betaine	0.78	0.75	0.75
Acetate	0.75	0.79	0.75
2-Hydroxybutyrate	0.72	0.68	0.91
Capric acid	0.71	0.76	0.70
Hypoxanthine	0.69	0.65	0.59
Cadaverine	0.65	0.61	0.93
Creatine	0.64	0.79	0.77
Creatinine	0.59	0.59	0.84
Butyrate	0.49	0.46	0.62
Threonine	0.43	0.45	0.76
Histidine	0.42	0.74	0.67

Glucose	0.39	0.37	0.62
O-Acetylcholine	0.31	0.48	0.43
Choline	0.29	0.32	0.50
Phenylalanine	0.28	0.57	0.65
2-Oxoglutarate	0.22	0.31	0.38
Lactate	0.21	0.33	0.38
Succinate	0.17	0.26	0.45
Formate	0.15	0.30	0.40
Malonate	0.13	0.54	0.58
3-Hydroxybutyrate	0.11	0.15	0.33
Isobutyrate	0.10	0.56	0.55
Acetone	0.10	0.15	0.33
3-Methyl-2-oxovalerate	0.09	0.40	0.43
Arginine	0.06	0.15	0.64
O-Acetylcarnitine	0.03	0.04	0.26
2-Oxoisocaproate	0.03	0.45	0.46
Methionine	0.02	0.49	0.58
3-Hydroxyisobutyrate	0.01	0.23	0.21
Proline	0.01	0.54	0.49

Variable importance (VIP) score for all 55 serum metabolites analyzed via PLS-DA. Metabolites with VIP ≥ 1.2 (shaded in gray) are considered to contribute significantly to the separate group clustering observed in the PLS-DA scores plots shown in figure 1 (of the main text).

Table S5. Variable Importance in Projection (VIP) scores associated with components 1, 2, and 3 of the PLS-DA model generated from the distinct stool metabolite profiles of the FD patients and healthy control relatives.

Metabolite	Comp. 1	Comp. 2	Comp. 3
Choline	3.43	2.36	2.29
Malonate	2.61	1.67	1.43
Tyramine	1.72	1.09	0.93
Beta-Alanine	1.66	1.02	0.92
p-Cresol	1.59	0.97	0.86
Phenylacetic acid	1.46	0.89	0.94
Lactate	1.42	0.89	0.81
Taurine	1.39	0.92	0.78
Ornithine	1.39	1.00	0.86
Isovalerate	1.31	0.80	0.79
Threonine	1.29	0.95	1.23
5-Aminopentanoate	1.26	0.95	1.02
Indole	1.24	0.94	1.12
Valerate	1.23	1.14	1.03
Glycerol	1.22	1.39	1.18
Isocaproate	1.22	0.85	1.05
Isobutyrate	1.19	0.91	0.92
Histidine	1.15	0.86	0.73
Dopamine	1.12	0.72	0.61
Xanthine	1.11	1.58	1.51
Inosine	1.11	1.41	1.27
Tryptophan	1.05	1.05	0.90
Formate	1.03	0.76	0.79
Valine	1.02	1.00	0.87
Orotic acid	1.01	1.21	1.03
Xylose	0.95	0.60	1.17
Trimethylamine	0.93	0.95	0.91
2-Aminobutyrate	0.93	0.96	0.99
Acetate	0.90	0.98	0.84
Glycerophosphocholine	0.89	0.70	0.69
Leucine	0.85	1.05	0.90
Ethanol	0.84	0.60	0.52
Phenylalanine	0.80	1.05	0.89
Malic acid	0.80	1.22	1.12
D-Mannose	0.79	0.95	1.06
Methylamine	0.79	1.36	1.29
Succinate	0.74	0.75	0.66

Glutamate	0.71	0.61	0.53
4-Hydroxyphenylacetate	0.71	0.68	0.70
Methylsuccinate	0.70	0.99	1.00
Glycine	0.68	0.59	0.53
Isoleucine	0.67	1.02	0.91
D-Glucose	0.67	0.41	1.63
Glucitol	0.65	0.94	0.85
Urocanate	0.64	1.35	1.56
Alanine	0.62	1.09	0.95
Aspartate	0.61	1.02	0.87
Lysine	0.59	1.01	0.90
Uracil	0.57	1.25	1.31
1,3-Dihydroxyacetone	0.55	0.85	1.00
Tyrosine	0.50	1.11	0.96
Thymine	0.45	0.52	1.32
Arabinose	0.45	0.70	0.93
Propionate	0.44	0.81	0.71
Dimethylamine	0.42	0.44	0.39
Proline	0.41	0.87	0.81
N-Acetylglutamic acid	0.41	1.08	1.07
2'-Deoxyuridine	0.36	1.23	1.07
Phosphoenolpyruvic acid	0.35	0.48	0.54
Fumarate	0.34	0.61	1.07
1,1-Dimethylbiguanide	0.31	0.42	0.36
Sarcosine	0.31	0.47	0.95
Uridine	0.29	1.33	1.19
Methionine	0.28	1.20	1.02
Glutamine	0.25	1.05	0.94
Glutarate	0.18	1.01	0.86
Nicotinate	0.16	0.81	0.83
Guanosine	0.15	0.79	0.68
Ribose	0.13	1.02	1.04
Arginine	0.11	0.60	0.54
Butyrate	0.07	0.94	0.81
2-Aminoisobutyric acid	0.04	0.96	1.09
Galactose	0.03	0.63	0.93

Variable importance (VIP) score for all 74 stool metabolites analyzed via PLS-DA. Metabolites with VIP ≥ 1.2 (shaded in gray) are considered to contribute significantly to the separate group clustering observed in the PLS-DA scores plots shown in figure 1 (of the main text).

Costello, Cheney et al., Supplementary Figure S1

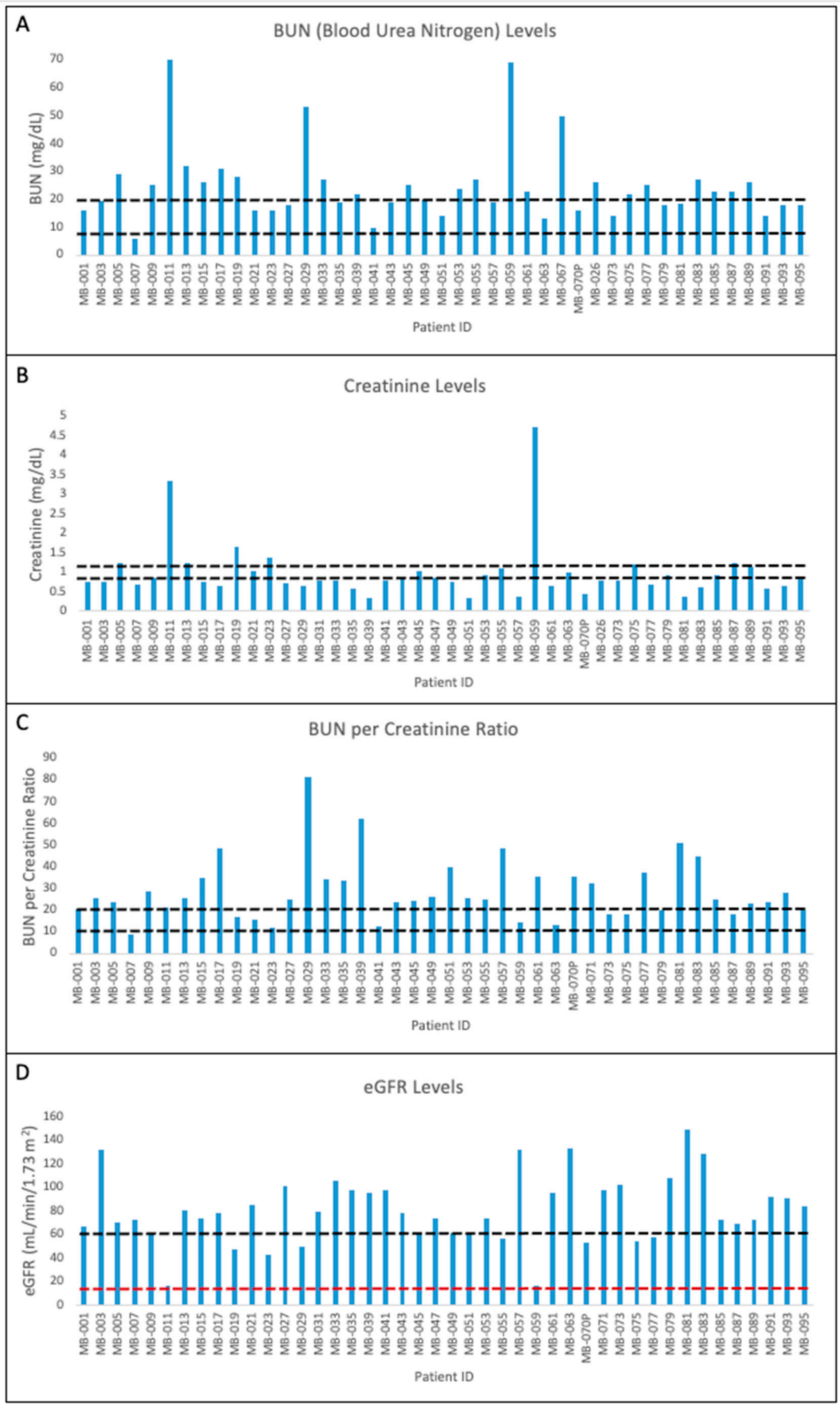

Figure S1. Results from Clinical Blood Tests that measured **(A)** blood urea nitrogen (BUN) levels, **(B)** creatinine levels, **(C)** BUN per creatinine ratio, and **(D)** estimated glomerular filtration rate (eGFR) levels in de-identified FD patient samples. These blood tests assessed the kidney function of FD patients via analysis of blood serum samples. **(A-C)** Levels within the dashed lines were considered normal. In **(D)**, for eGFR levels, levels below the black dashed line suggested chronic kidney disease, and levels below the red dashed line suggests kidney failure. Above the black dashed line indicates clinically normal levels (Mayo Clinic).

Costello, Cheney et al., Supplementary Figure S2

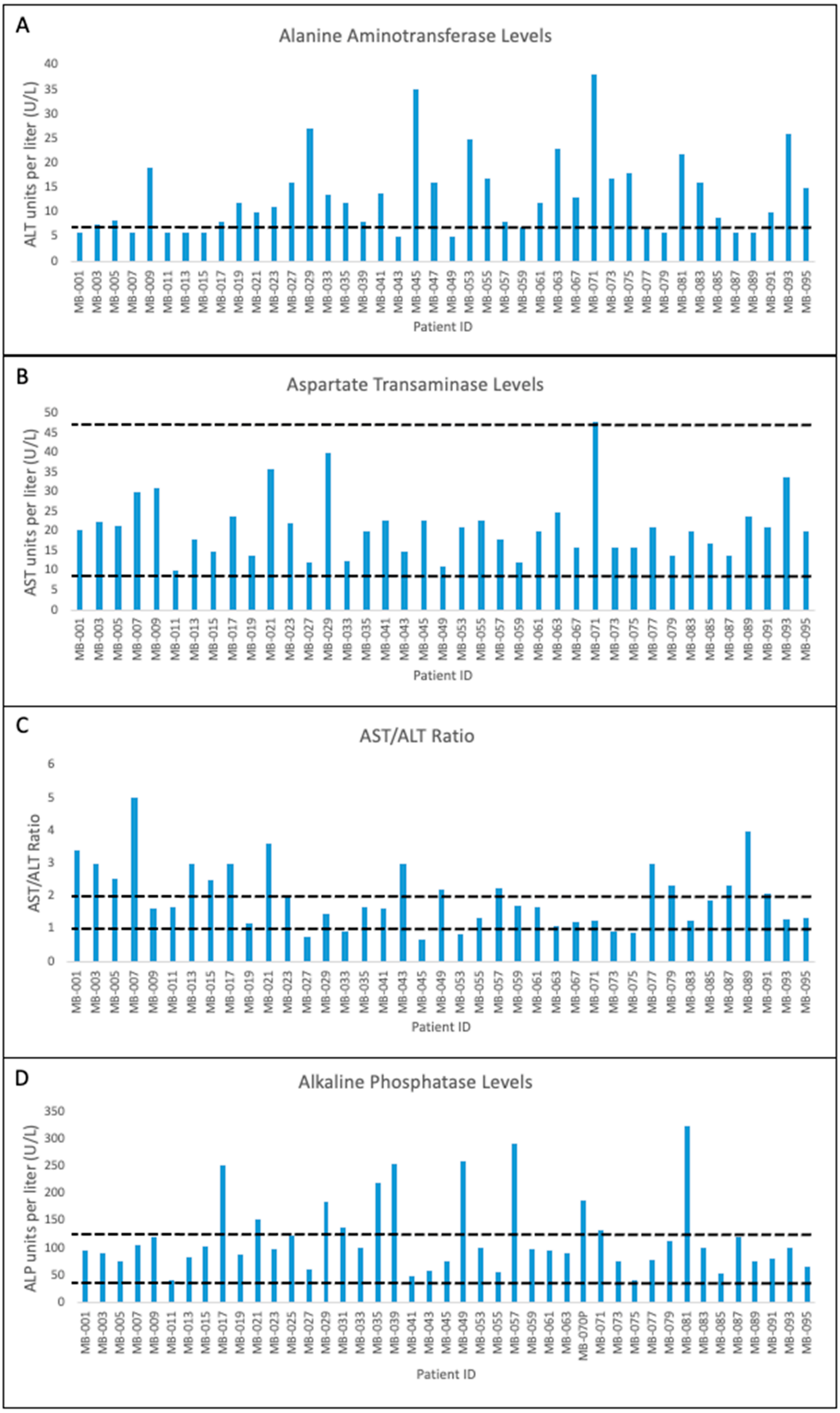

Figure S2. Results from FD patient liver enzyme blood tests and measurements of **(A)** Alanine aminotransferase (ALT) levels; **(B)** aspartate transaminase (AST) levels; **(C)** AST/ALT ratio; and **(D)** alkaline phosphatase levels (ALP). These blood tests assessed liver function by measuring the levels of liver enzymes in blood serum samples of FD patients. In panel **(A)**, values above the dashed line are considered normal ALT levels. In panels **(B-D)**, values between the dashed lines are considered clinically normal (Mayo Clinic). These data were collected on de-identified patient serum samples by clinical staff of the Familial dysautonomia center, at New York University School of Medicine.

Costello, Cheney et al., Supplementary Figure S3

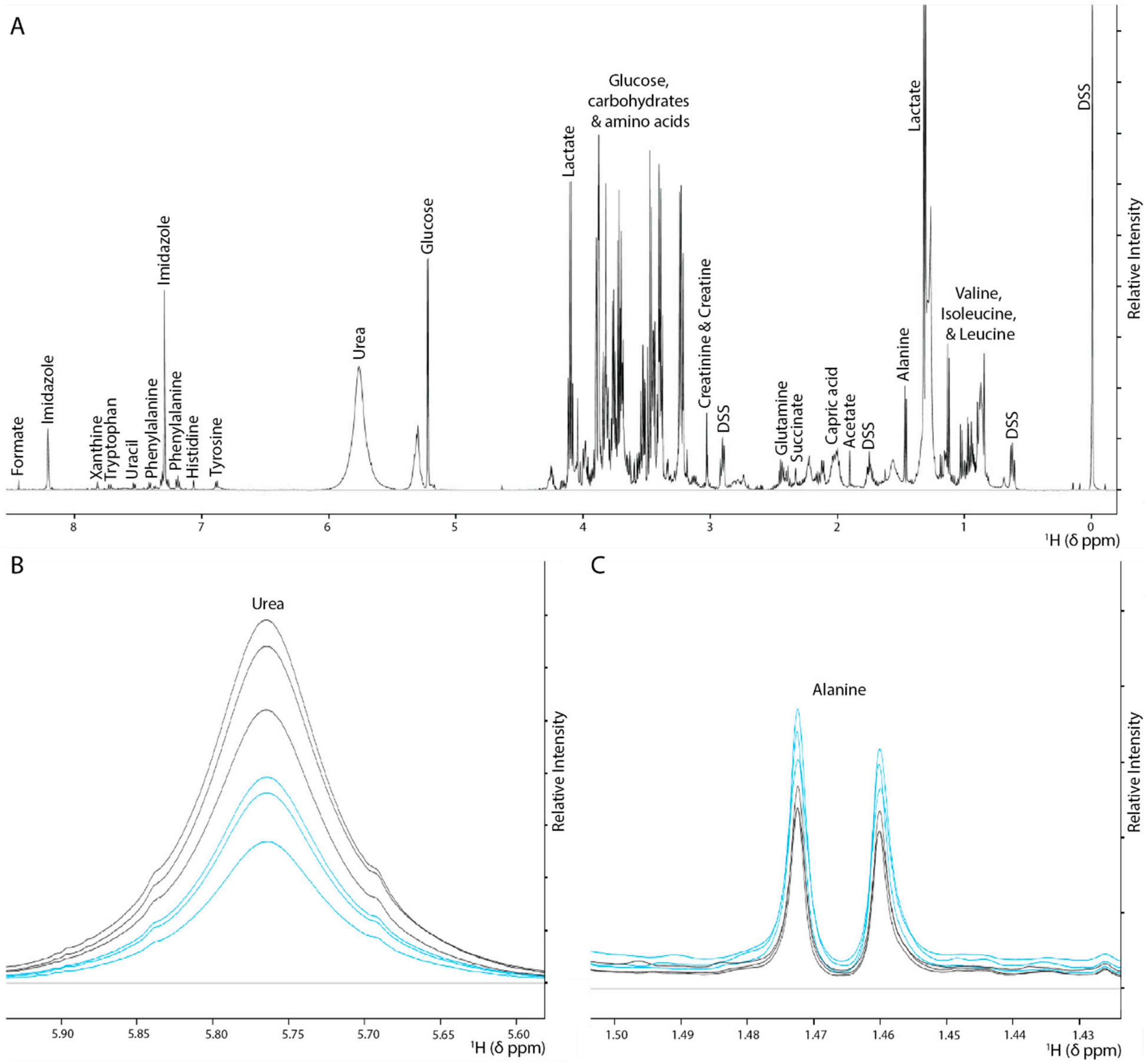

Figure S3. Human serum mixed metabolite 1D ^{1}H-NMR (600 MHz) spectra. **(A)** Overlays of 1D ^{1}H NMR spectra highlighting metabolites identified in serum metabolite mixtures of FD patients and healthy relative controls. Underlined metabolites denote those with multiple signals throughout the 1H chemical shift range. Overlays of ^{1}H NMR spectra from FD patient serum samples (black) and control relative serum samples (blue) are shown in **(B)** for the chemical shift region 5.6-5.9 ppm corresponding to urea, and in **(C)** for the chemical shift region 1.45-1.48 ppm corresponding to alanine.

Costello, Cheney et al., Supplementary Figure S4

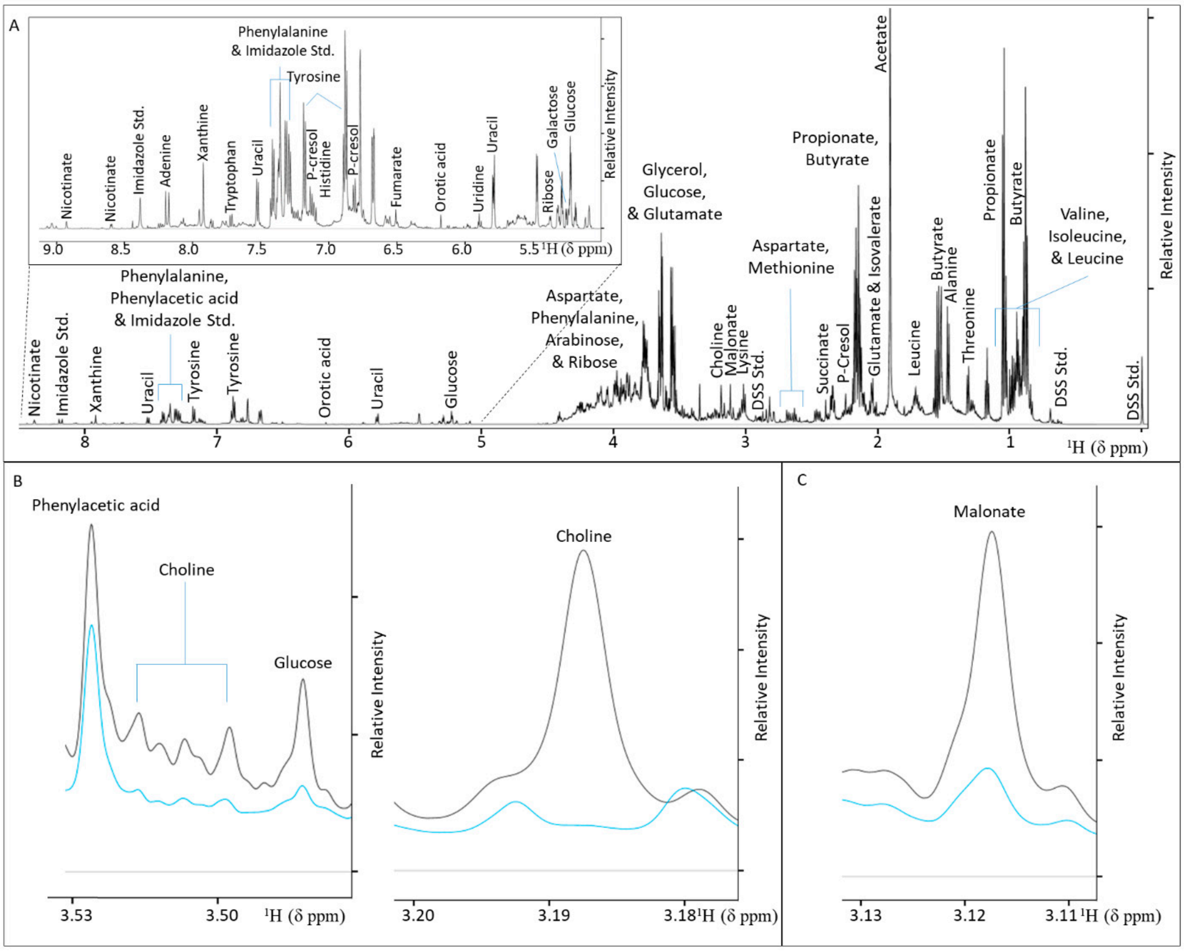

Figure S4. Human fecal metabolite 1D ^{1}H-NMR spectra. **(A)** Overlays of 1D 1H NMR spectra highlighting metabolites identified in the stool metabolite extracts of FD patients and healthy relative controls. Overlays of 1H NMR spectra from FD patient (black) and healthy relative control (blue) stool samples (black) are shown in **(B)** for choline, at chemical shift range 3.18-3.20 ppm, and 3.50-3.53 ppm, and **(C)** for malonate at 3.11-3.13 ppm. Additional peaks (phenylacetic acid and glucose) are annotated in spectral ranges as they appear in **(B)**.

Costello, Cheney et al. Supplementary Figure S5

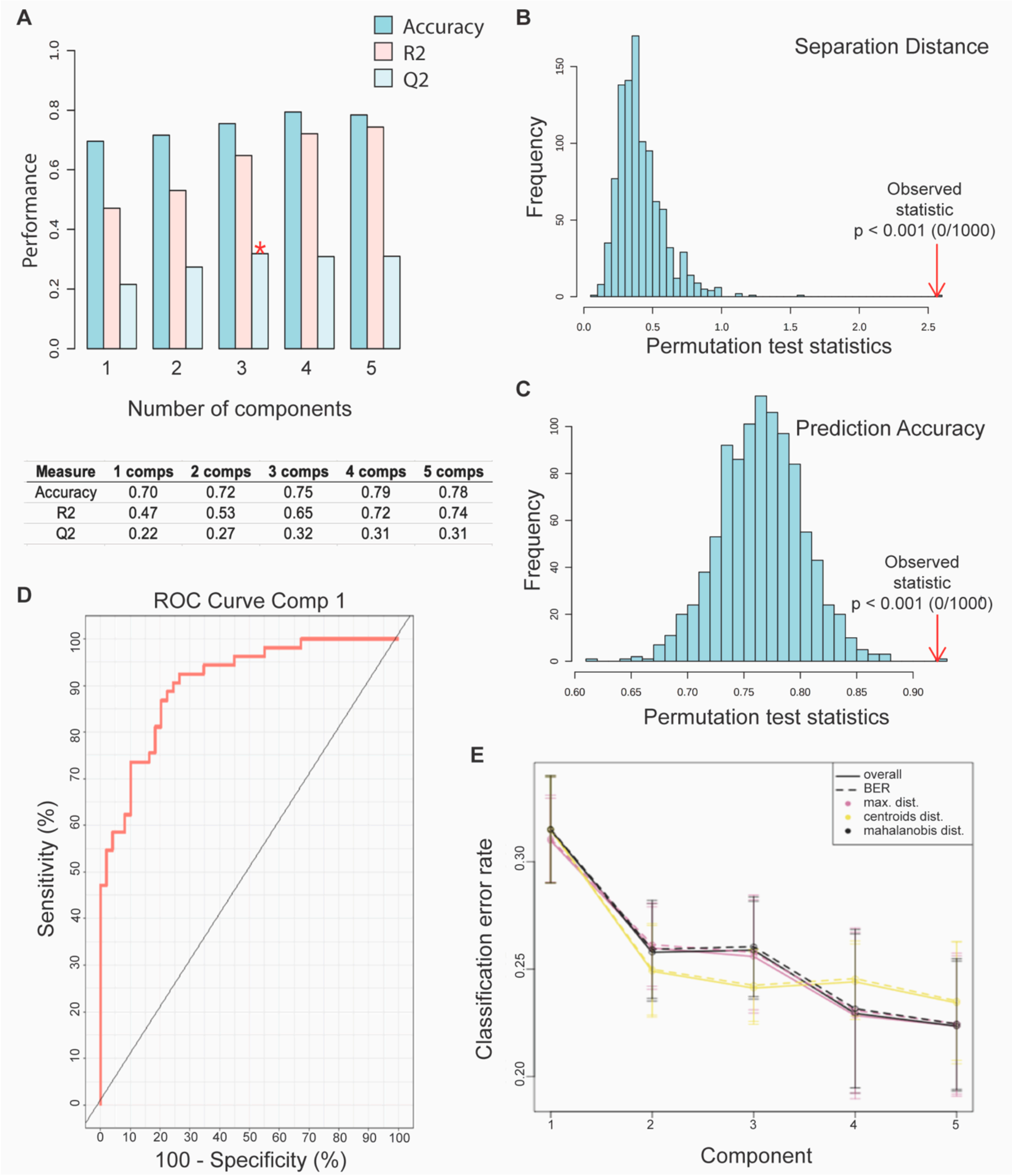

Measure	1 comps	2 comps	3 comps	4 comps	5 comps
Accuracy	0.70	0.72	0.75	0.79	0.78
R2	0.47	0.53	0.65	0.72	0.74
Q2	0.22	0.27	0.32	0.31	0.31

Figure S5. Validation metrics associated with the PLS-DA modeling of serum metabolite profiles of FD patients and healthy controls. **(A)** Leave-one-out cross validation (LOOCV) plot demonstrated model significance with an accuracy score of 0.75, an R^2 of 0.65, and Q^2 of 0.32, when taking into account the first 3 components of the PLS-DA model. The separation distance **(B)** and prediction accuracy permutation tests **(C)** (n = 1000 tests) yielded significant p-values < 0.001, attesting to the validity and prediction accuracy of the PLS-DA model. **(D)** The area under receiver operating characteristic (AUROC) curves yielded an AUC value of 0.91, demonstrating high model accuracy and specificity for the separate classification of FD patient and healthy control groups. **(E)** Classification error rate (CER) yielded a remaining classification error rate of ~26% for component 3 and less than 25% for component 4 (and beyond) of the PLS-DA model.

Costello, Cheney et al. Supplementary Figure S6

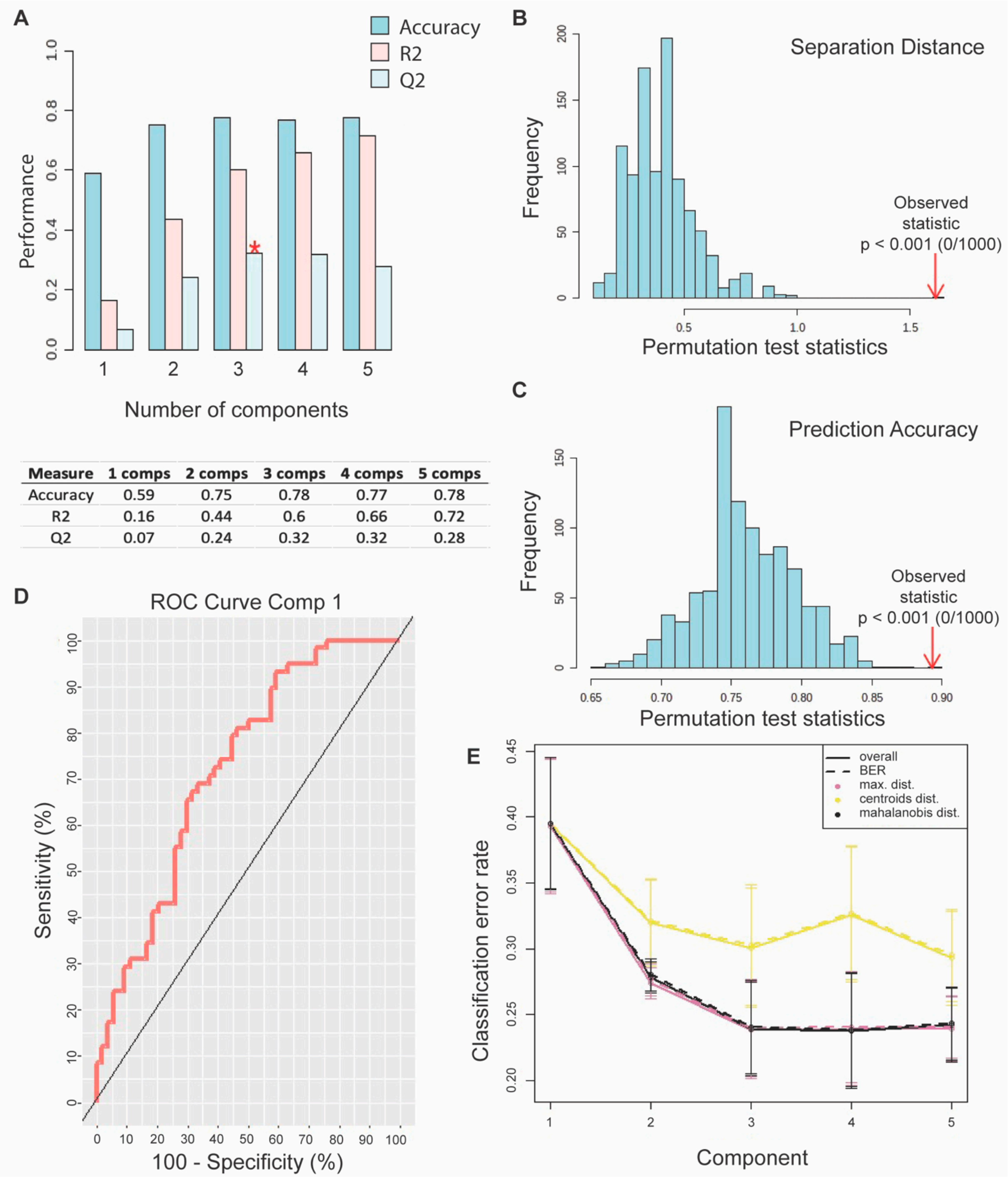

Measure	1 comps	2 comps	3 comps	4 comps	5 comps
Accuracy	0.59	0.75	0.78	0.77	0.78
R2	0.16	0.44	0.6	0.66	0.72
Q2	0.07	0.24	0.32	0.32	0.28

Figure S6. Validation Metrics associated with the PLS-DA modeling of stool metabolite profiles of FD patients and healthy controls. **(A)** Leave-one-out cross validation (LOOCV) plot demonstrated model significance with an accuracy score of 0.78, an R^2 of 0.6, and Q^2 of 0.32, when taking into account the first 3 components of the PLS-DA model. The separation distance **(B)** and prediction accuracy permutation tests **(C)** (n = 1000 tests) yielded significant p-values < 0.001, attesting to the validity and prediction accuracy of the PLS-DA model, demonstrating high model accuracy and specificity for the separate classification of FD patient and healthy control groups. **(D)** The area under receiver operating characteristic (ROC) curves yielded an AUC value of 0.72. **(E)** Classification error rate (CER) yielded a remaining classification error rate of ~25% for component 3 and less than 25% for component 4 (and beyond) of the PLS-DA model.

Costello, Cheney et al. Supplementary Figure S7

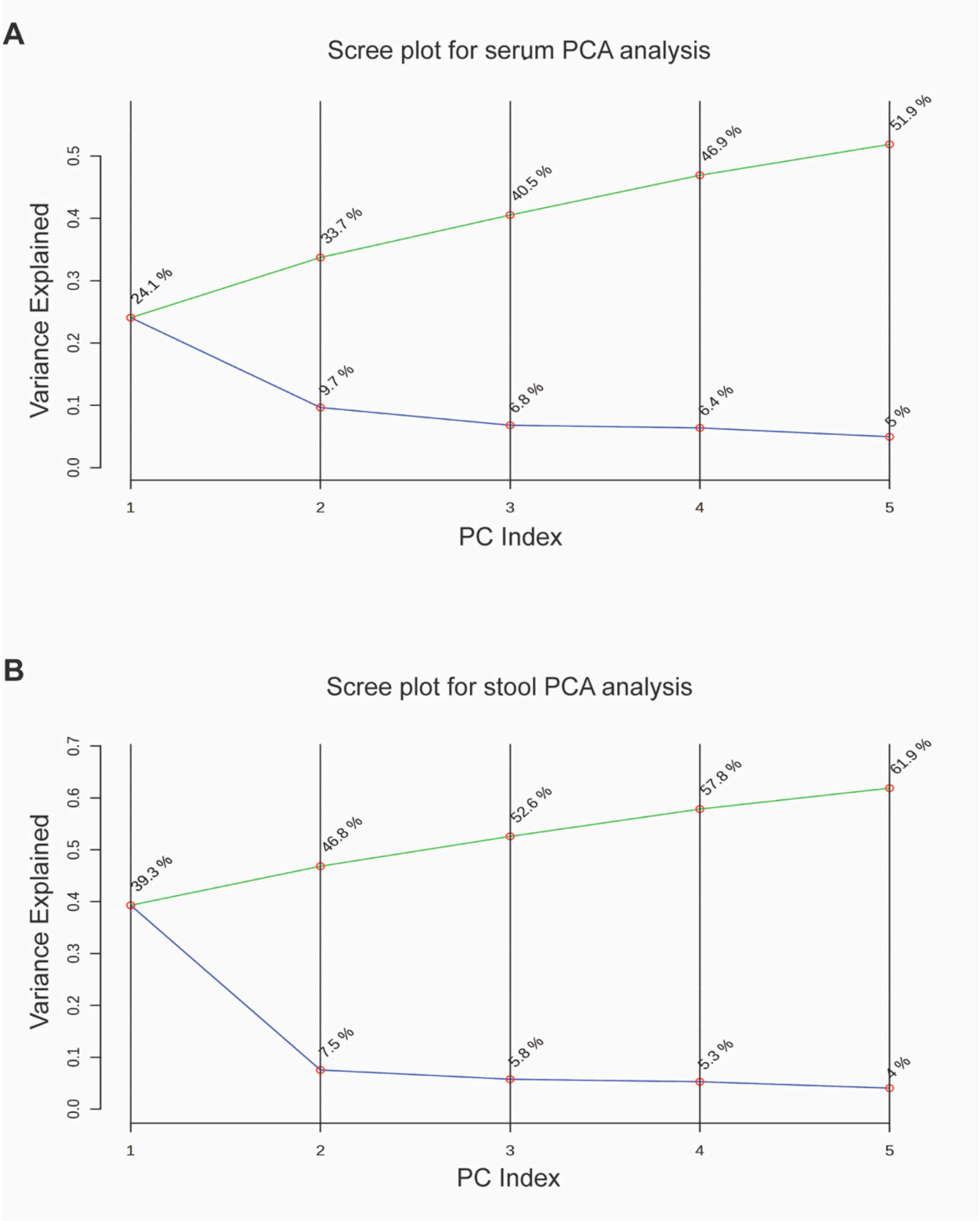

Figure S7. Scree plots associated with unsupervised PCA analyses of **(A)** serum and **(B)** stool metabolite profiles. The plots indicate the percent variance contribution of each principal component (from PC1 to PC5) of the PCA scores plots. The green line represents the total cumulative variance as the percent variance of each PC 1-5 is added. The blue line represents the percent variance accounted for by each PC. The scree plots were generated using the principal component analysis module of MetaboAnalyst v.4.0.

Costello, Cheney et al., Supplementary Figure S8

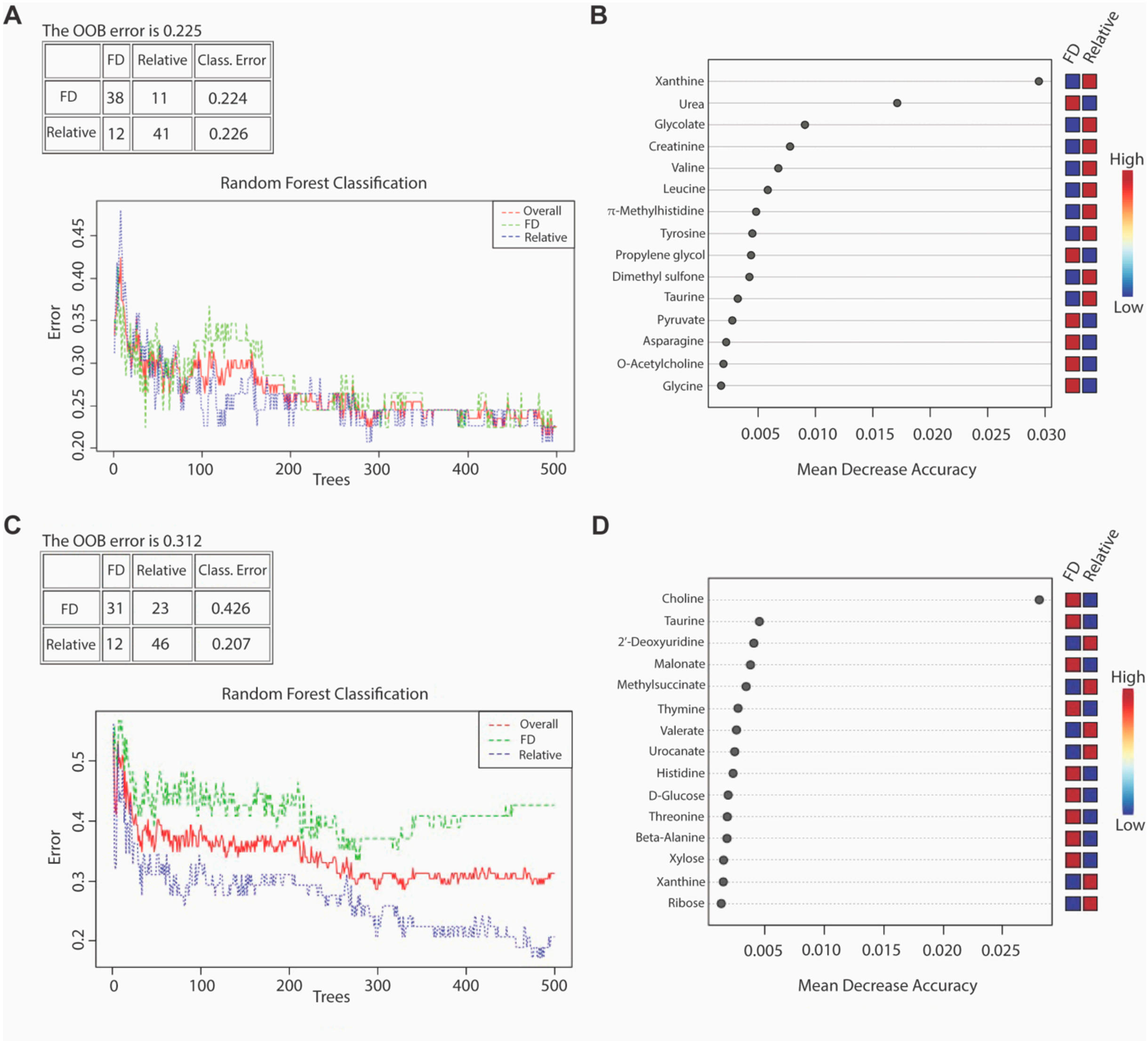

Figure S8. Random forest analysis of the FD patient vs Control relative groups. **(A)** The analysis for serum profiles yielded an overall out-of-bag (OOB) error of 0.225 (22.5%) and classification error rates of 0.224 (22.4%) and 0.226 (22.6%) for the FD and control groups, respectively**. (B)** Mean decrease accuracy values highlighted the top 15 serum metabolites that were found to be important discriminators of the two groups and contributed most to the accuracy of the random forest predictions. **(C)** For the stool profile analyses, the OOB error was 0.312 (31.2%), with the FD class error being 0.426 (42.6%) and the control class error being 0.207 (20.7%). **(D)** The top 15 metabolites of the mean decrease accuracy plot are listed, with their concentration trend in relation to the control group shown to the right. Blue and Red indicate a low and high concentration trend, respectively. Mean decrease accuracy is shown along the bottom.

The random forest analysis of the serum metabolite profiles yielded a comparable classification error rate to that observed in the CER evaluation of the first three components of the PLS-DA model (i.e., a CER value of ~25%) compared to an out-of-bag (OOB) error rate of 22.5% in the random forest analysis (Figures S1E & S3A). Similarly, the random forest analysis of the stool metabolite profiles identified an

overall OOB error rate of 31.2%, which is relatively consistent with a CER of ~25% (Figures S2E & S3C). Additionally, the random forest analysis of the stool metabolome yielded a more accurate classification of the control samples (class error = 20.7%) compared to those of the FD patients (class error = 42.6%), consistent with the greater dispersion observed within the FD patient cluster compared to the control group in the 3D PLS-DA scores plot shown in Figure 1D. This indicates that the stool metabolomes of FD patients are not only different from controls but vary significantly from each other.

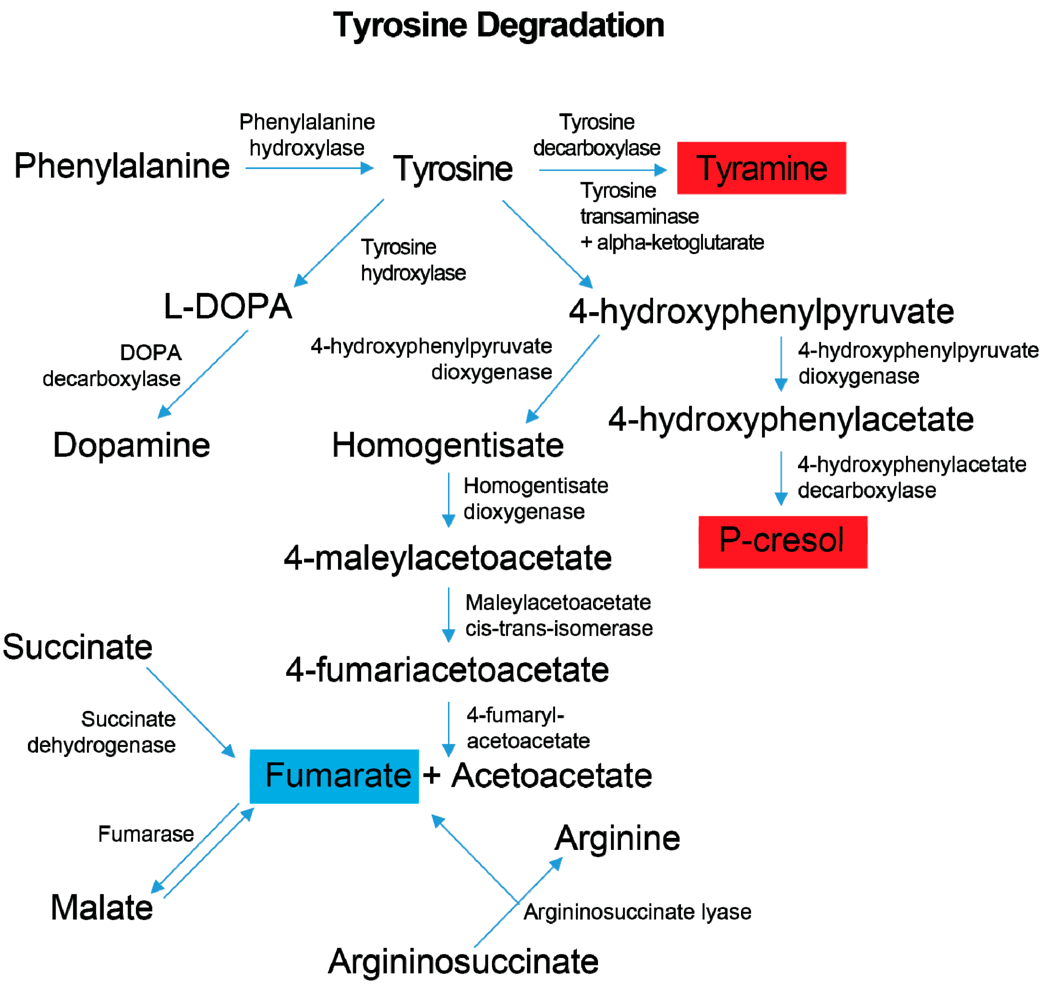

Figure S9. Tyrosine pathways yield tyramine, p-cresol, fumarate, and dopamine. The one-step reaction facilitated by tyrosine decarboxylase converts tyrosine to tyramine. Through a three-step reaction, tyrosine can also be converted to p-cresol. Lastly, fumarate and acetoacetate (linked to the TCA cycle) can be produced by a four-step reaction [1]. In addition to tyrosine metabolism, fumarate is classically produced by the TCA cycle from the conversion of succinate to fumarate via succinate dehydrogenase (Complex II of the ETC). In addition to the metabolites of interest from our study, dopamine, relevant for neurological signaling, is made from the starting substrate tyrosine. Red indicates metabolites with elevated levels in the FD patients stool samples, while blue indicates metabolites that were lower in concentration in the stool samples of FD patients.

Costello, Cheney et al., Supplementary Figure S10

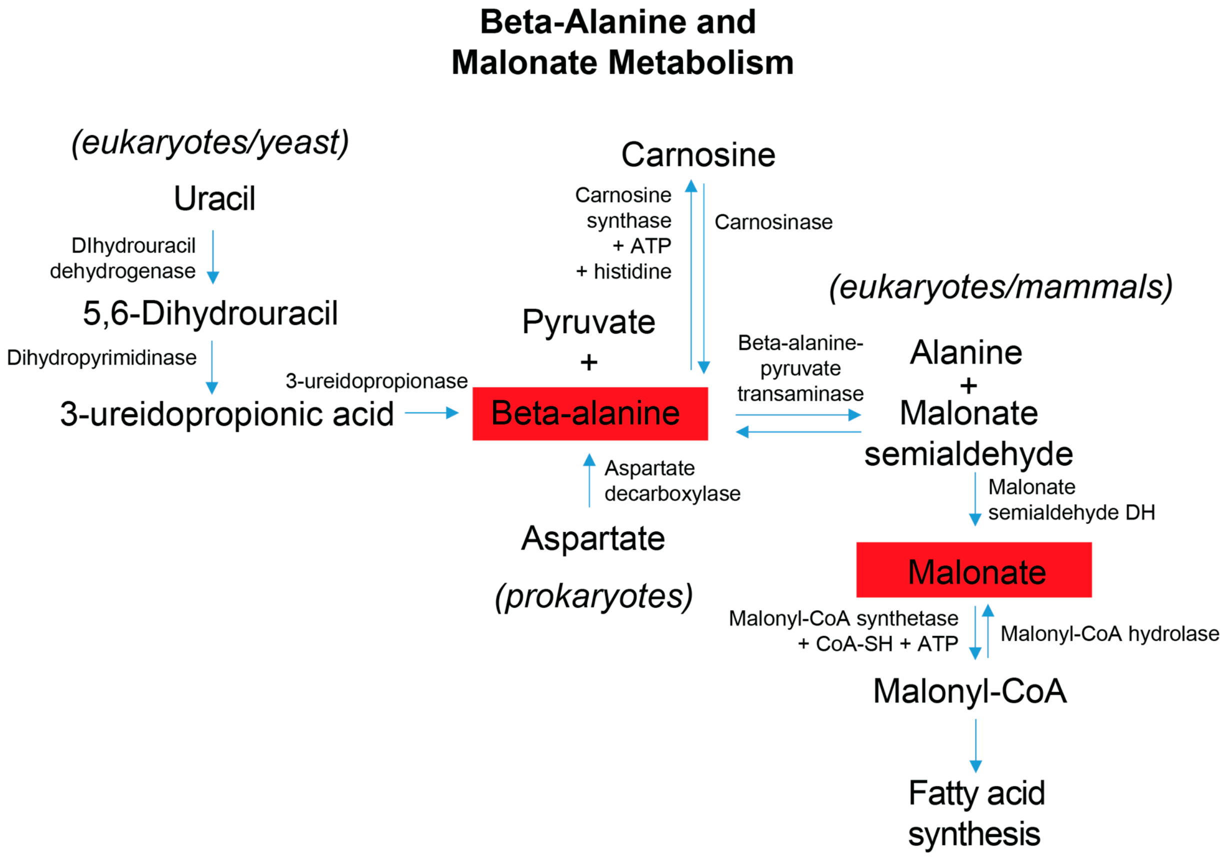

Figure S10. Beta-alanine and malonate metabolism connect via the intermediate malonate semialdehyde. Beta-alanine is produced by the degradation of uracil, decarboxylation of aspartate by gut microbes, carnosine catabolism, and a transaminase reaction using alanine and malonate semialdehyde as substrates [2, 3]. Carnosine is a constituent of muscle, and beta-alanine (in addition to histidine) are produced when muscle carnosine is broken down [4], which take place both when human skeletal muscle and food sources of muscle proteins are degraded. Red indicates metabolites with elevated levels in the FD patients stool samples. Abbreviations: ATP, adenosine triphosphate; DH, dehydrogenase; CoA, coenzyme-A.

Costello, Cheney et al., Supplementary Figure S11

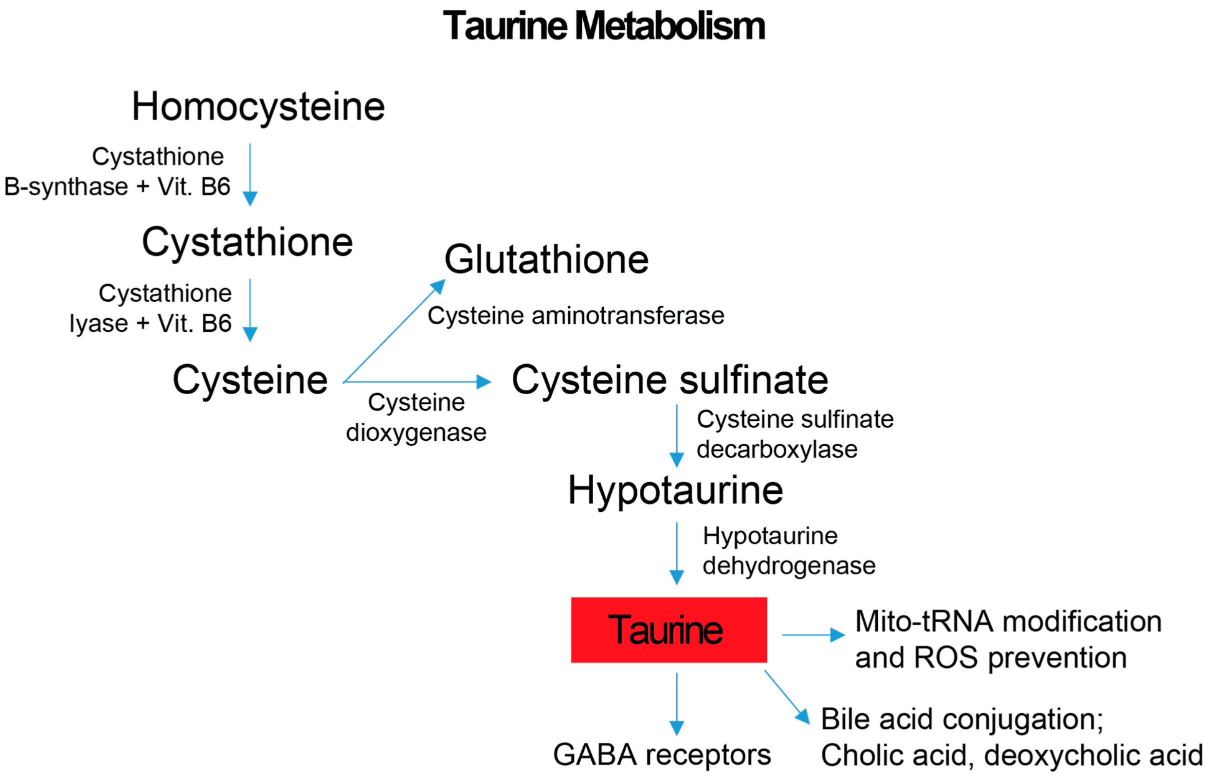

Figure S11. Taurine is obtained primarily from the diet but can also be synthesized endogenously via degradation of homocysteine and participates in 3 primary host metabolic functions. Taurine can conjugate to bile acids, specifically cholic and deoxycholic acid. Taurine functions in the nervous system as an inhibitory signaling molecule as discussed in Figure S13. Lastly, taurine conjugates to mitochondrial tRNA to aid in translation of mitochondrial proteins, specifically enhancing the production of ETC complex 1 protein components and mitigating the production of ROS by facilitating proper complex 1 protein subunit production, and therefore ETC function [5]. Red indicates taurine was elevated in FD patients stool samples. Abbreviations: GABA, gamma-aminobutyric acid; Mito, mitochondria; ETC, electron transport chain; ROS, reactive oxygen species.

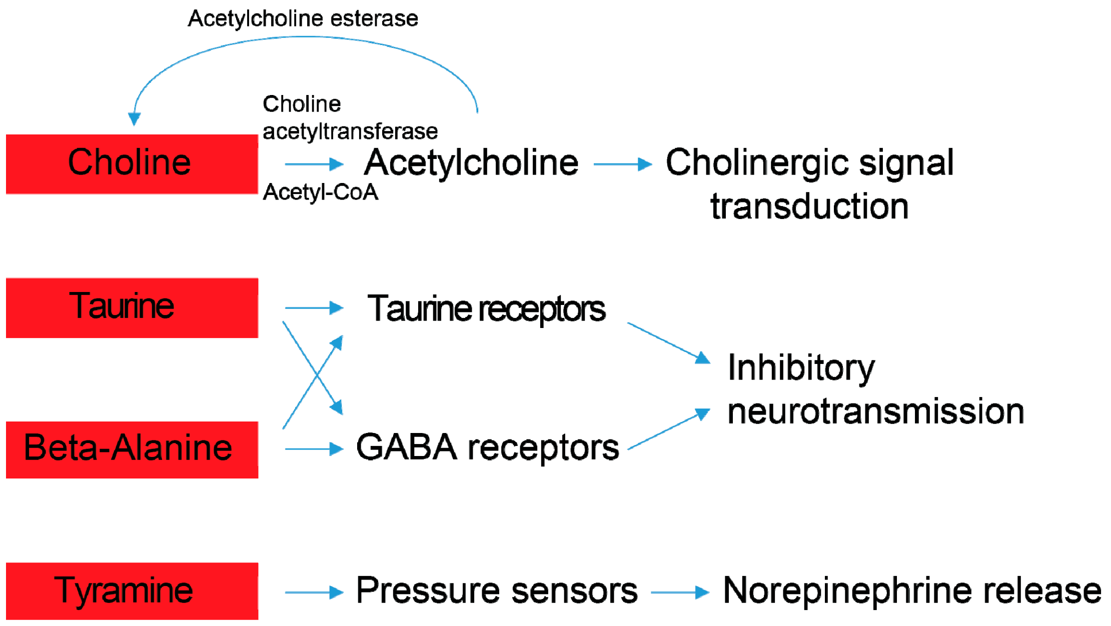

Figure S12. The four neural signaling molecules identified in elevated concentrations in the stool of FD patients compared to their control relatives. Choline is converted to acetylcholine via a one-step reaction requiring acetyl-CoA; this reaction occurs in the pre-synaptic neuron and the product (acetylcholine) is exported to the synaptic cleft of cholinergic neurons where it can bind postsynaptic muscarinic and preganglionic nicotinic receptors [6, 7]. Intra-synaptic acetylcholinesterase terminates the signal and liberates choline from acetylcholine to recycle some choline back into the neuron. Taurine and beta-alanine can both bind GABA receptors to initiate an inhibitory neural signal. Additionally, taurine can elicit neuronal hyperpolarization through its own unique signaling system[8], whereas beta-alanine acts primarily as a “false neurotransmitter”, binding GABA receptors and inducing an improper inhibitory response[4]. Tyramine binds pressure sensors and promotes norepinephrine release. Red indicates metabolites in higher concentrations in FD patients stool samples. Abbreviations: GABA, gamma-aminobutyric acid

Supplementary References Cited

1. Stančíková, M., and Rovenský, J. "Metabolism of Aromatic Amino Acids." In *In Alkaptonuria and Ochronosis*, edited by J. Rovensky, Urbanek, T, Boldisova, O., and Gallagher, J., 9-12. Slovakia: Springer, 2015.
2. Eaton, K.K., Howard M., and Hunnisett, A. "Urinary Beta-Alanine Excretion Is a Marker of Abnormal as Well as Normal Gut Fermentation." *J. Nutr. Environ. Medicine* 4, no. 2 (1994): 157-63.
3. Lopez-Samano, M., Lozano-Aguirre Beltran, L.F., Sanchez-Thomas, R., Davalos, A., Villasenor, T., Garcia-Carcia, J.D., Garcia-de los Santos, A. "A Novel Way to Synthesize Pantothenate in Bacteria Involves B-Alanine Synthase Present in Uracil Degradation Pathway." *MicrobiologyOpen* 9(4):e1006 (2020).
4. Tiedje, K. E., K. Stevens, S. Barnes, and D. F. Weaver. "B-Alanine as a Small Molecule Neurotransmitter." *Neurochemistry International* 57, no. 3 (2010): 177-88.
5. Jong, C. J., J. Azuma, and S. Schaffer. "Mechanism Underlying the Antioxidant Activity of Taurine: Prevention of Mitochondrial Oxidant Production." *Amino Acids* 42, no. 6 (2012): 2223-32.
6. Wiedeman, A. M., S. I. Barr, T. J. Green, Z. Xu, S. M. Innis, and D. D. Kitts. "Dietary Choline Intake: Current State of Knowledge across the Life Cycle." *Nutrients* 10, no. 10 (2018).
7. Wurtman, Richard J. "Choline Metabolism as a Basis for the Selective Vulnerability of Cholinergic Neurons." *Trends in Neurosciences* 15, no. 4 (1992): 117-22.
8. Wu, J. Y., and H. Prentice. "Role of Taurine in the Central Nervous System." *J Biomed Sci* 17 Suppl 1 (2010): S1.
